# Eupalinolide B suppresses pancreatic cancer by ROS generation and potential cuproptosis

**DOI:** 10.1016/j.isci.2024.110496

**Published:** 2024-07-14

**Authors:** Qingtian Huang, Jie Yang, Jiaxing Zhang, Leyi Yao, Baoyi Jiang, Siyuan Du, Fengjin Li, Qian Peng, Lingsha Qin, Yanfen Wang, Ling Qi

**Affiliations:** 1 Institute of Digestive Diseases, the Affiliated Qingyuan Hospital (Qingyuan Peoples's Hospital), Guangzhou Medical University, Qingyuan 511518, Guang Dong, China; 2 Biological Sample Resource Centre, the Affiliated Qingyuan Hospital (Qingyuan Peoples's Hospital), Guangzhou Medical University, Qingyuan 511518, Guang Dong, China; 3 Department of Pathology, the Affiliated Qingyuan Hospital (Qingyuan Peoples's Hospital), Guangzhou Medical University, Qingyuan 511518, Guang Dong, China; 4 Division of Gastroenterology, Institute of Digestive Disease, the Affiliated Qingyuan Hospital (Qingyuan Peoples's Hospital), Guangzhou Medical University, Qingyuan 511518, Guang Dong, China

**Keywords:** Molecular biology, Cell biology, Cancer

## Abstract

Pancreatic cancer is highly lethal with limited effective treatments. This study explores the therapeutic effects of eupalinolide B (EB) from *Eupatorium lindleyanum* DC on pancreatic cancer cells. Through cellular functional assays, we observed that EB effectively inhibits cell viability, proliferation, migration, and invasion. In a xenograft mouse model, EB treatment resulted in reduced pancreatic cancer tumor growth and decreased expression of Ki-67. Mechanistically, EB induces apoptosis, elevates reactive oxygen species (ROS) levels, and disrupts copper homeostasis. RNA sequencing (RNA-seq) and gene set enrichment analysis (GSEA) identified copper ion binding pathways and potential involvement in cuproptosis. Furthermore, EB enhances the cytotoxic effects of elesclomol (ES), increasing ROS levels in a copper-dependent manner and exhibiting synergistic cytotoxicity. These findings suggest that EB, either alone or in combination with ES, represents a promising strategy for targeting metal ion dysregulation and inducing potential cuproptosis in pancreatic cancer, offering a potential improvement in therapeutic outcomes.

## Highlights

• EB inhibits pancreatic cancer cells growth
• EB induces ROS generation and potential cuproptosis
• EB has a synergistic effect with ES in inducing cuproptosis in pancreatic cancer cells

## Graphical abstract

**Figure:**
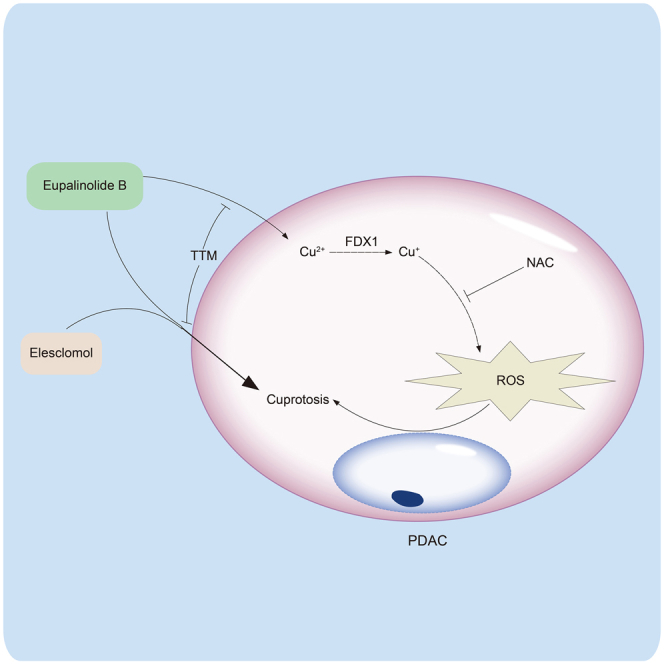

## Introduction

Pancreatic cancer is widely recognized as a highly lethal malignancy characterized by low survival rates and ineffective treatment options. Its status as one of the deadliest cancers globally is deeply concerning. Recent data indicate that the incidence rates for both men and women are steadily increasing at a rate of approximately 1% per year. Consequently, pancreatic cancer currently holds the unenviable distinction of having the worst five-year survival rate, standing at a mere 12%.^1^ Pancreatic cancer is often diagnosed at an advanced stage, largely due to the pancreas’s concealed anatomical position and the lack of distinctive symptoms in the early stages. At the time of diagnosis, the majority of patients present with either locally advanced (30%–35%) or metastatic disease (50%–55%).^2^ Late-stage diagnosis often precludes surgery, the most effective treatment option, in many cases. Even among patients who undergo surgical intervention, the recurrence rate of pancreatic cancer remains high, with rapid progression of metastatic disease limiting treatment options. Traditional treatment strategies for pancreatic cancer include surgery, radiation therapy, chemotherapy, and more recently, targeted and immunotherapies.^3^ While these treatments have been effective for other cancer types, their efficacy is limited in pancreatic cancer. Surgical resection is typically only viable for early-stage cases, with most patients being diagnosed beyond the operable phase. Radiation and chemotherapy, used as adjuvant or palliative treatments, often come with severe side effects and have limited impact on extending survival. Additionally, the high adaptability and resistance of pancreatic cancer cells cast doubt on the long-term effectiveness of these traditional treatments.

Amid the limitations of conventional treatments, herbal extracts, with their unique mechanisms, lower toxicity, and multi-target effects, are gaining widespread attention. Recent advances in herbal extract research in oncology, particularly for intractable cancers, have highlighted their potential benefits. Studies into the material basis and mechanisms of action of herbal extracts not only enrich the modern pharmacological theories of traditional Chinese medicine but may also provide additional avenues for cancer treatment. Many clinically used drugs, such as paclitaxel and camptothecin, are derived directly or indirectly from natural products.^4^^,^^5^ As research progresses, the anti-tumor mechanisms, active compounds, and target sites of herbal extracts are being revealed, offering hope for the treatment of pancreatic cancer. *Eupatorium lindleyanum* DC (EL), also known as Ye Ma Zhui in traditional Chinese medicine, is credited with expectorant, antitussive, anti-asthmatic, heat-clearing, detoxifying, diuretic, edema-relieving, and antihypertensive properties. It is particularly effective for chronic bronchitis and bronchitis. Clinical products include Ye Ma Zhui tablets and syrup. Due to its low toxicity, recent studies on the chemical components and pharmacological activities of EL have unveiled its significant medicinal potential in anti-tumor,^6^^,^^7^^,^^8^^,^^9^^,^^10^ anti-inflammatory,^11^ antioxidant activities,^12^ and respiratory system^13^^,^^14^ protection. Eupalinolide B (EB), an active constituent isolated from EL, has demonstrated potential therapeutic effects on various cancers such as lung and liver cancer; however, its efficacy in pancreatic cancer remains unclear.

The recent discovery of cuproptosis, a distinct form of programmed cell death driven by copper overload, has unveiled pathways separate from known mechanisms such as apoptosis, necrosis, or autophagy. Cuproptosis is primarily initiated by the binding of copper ions to mitochondrial lipoylated proteins, leading to their aggregation and subsequent destabilization of iron-sulfur cluster proteins. This disruption impairs the mitochondrial electron transport chain, culminating in cell death.^15^

The potential for cuproptosis to serve as an anticancer strategy is particularly promising for targeting hard-to-treat tumors, such as pancreatic cancer, especially in cases where cells have developed resistance to chemotherapy and radiotherapy. By manipulating copper metabolism, it is possible to selectively induce cuproptosis in cancer cells, thereby inhibiting their growth and survival. The role of copper transport proteins and copper-dependent enzyme systems is crucial in this process, offering various targets for therapeutic drug development.^16^ Copper stress not only couples with cuproptosis, but also leads to reactive oxygen species (ROS) stress, oxidative damage and cell-cycle arrest.^17^

Our study demonstrates that EB can increase the accumulation of copper ions in pancreatic cancer cells, promoting cuproptosis and consequently inhibiting cell proliferation. This not only underscores the potential of cuproptosis in pancreatic cancer treatment but also provides experimental evidence for integrating traditional Chinese medicine into modern oncology. Elesclomol (ES), a known copper ionophore, facilitates the transport of copper ions into mitochondria,^18^ enhancing copper concentration and inducing oxidative stress-dependent cuproptosis. Both *in vitro* and preclinical studies have shown that ES effectively induces cancer cell death, especially when used in conjunction with additional copper ions. ES-mediated copper delivery involves both ferredoxin 1 (FDX1)-dependent and independent mechanisms. FDX1 plays a pivotal role in the mitochondrial reduction of Cu^2+^ to Cu^+^, essential for the activation of cuproptosis. However, ES can also deliver copper to other cellular compartments, contributing to its cytotoxic effects. This broader copper release, not limited to mitochondria, expands the potential therapeutic applications of ES.^18^ Within this context, our findings suggest that EB enhances the anticancer activity of ES through mechanisms involving cuproptosis. The combined use of EB and ES could potentiate the induction of cuproptosis, thereby increasing the therapeutic efficacy of ES in treating cancer.

This article aims to unveil the molecular mechanisms of EB, investigating how it modulates the copper to induce cuproptosis in pancreatic cancer cells and to develop adjuvant therapies for pancreatic cancer treatment. Furthermore, EB sensitizes ES-induced cuproptosis in pancreatic cancer cells, offering perspectives and strategies for treatment, and enhances our understanding of cuproptosis with significant implications for cancer biology and therapeutic research.

## Results

### EB exhibits inhibitory effects on the biological functions of pancreatic cancer cells

**Figure 1:**
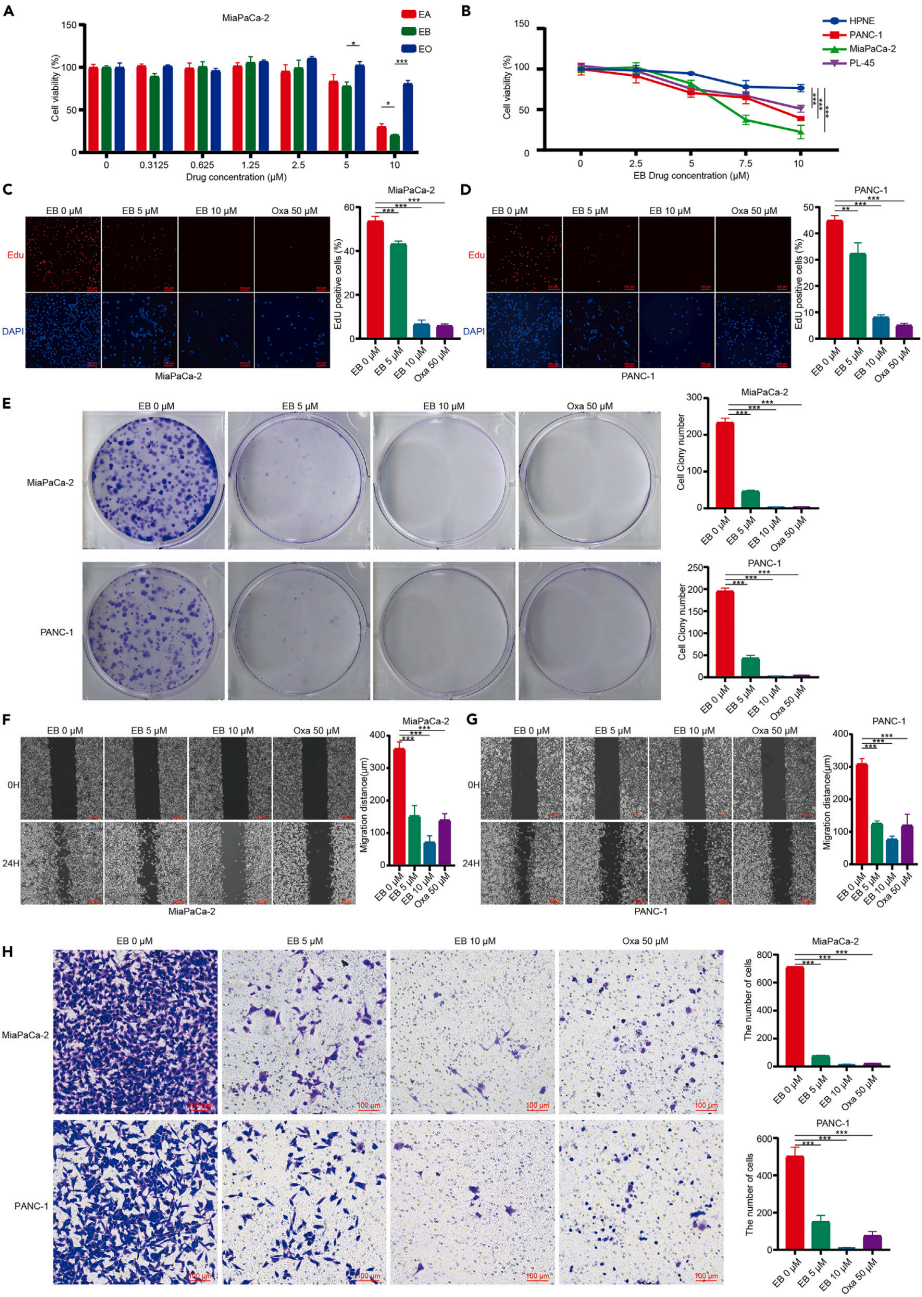
EB exhibits inhibitory effects on the biological functions of pancreatic cancer cells (A) The effect of different concentrations of EA, EB, and EO on the viability of MiaPaCa-2 pancreatic cancer cells. (B) The impact of various concentrations of EB on the viability of HPNE normal pancreatic cells and PANC-1, MiaPaCa-2, PL-45 pancreatic cancer cells. (C) Left: Edu fluorescence staining of MiaPaCa-2 cells after treated with varying concentrations of EB or Oxa. Right: the statistical chart of the percentage of Edu-positive cells. (D) Left: Edu fluorescence staining of PANC-1 cells post-treatment with varying concentrations of EB or Oxa. Right: the statistical chart of the percentage of Edu-positive cells. (E) Left: the clone colony formation of pancreatic cancer cells after treatment with varying concentrations of EB or Oxa. Right: the statistical chart of the number of clone colonies. (F) Left: the wound healing assay of MiaPaCa-2 cells treated with different concentrations of EB or Oxa. Right: the statistical chart of the wound closure distance. (G) Left: the wound healing assay of PANC-1 cells treated with different concentrations of EB or Oxa. Right: the statistical chart of the wound closure distance. (H) Left: The invasion of pancreatic cancer cells after treatment with different concentrations of EB or Oxa. Right: the associated statistical chart. Three replicates per group were set up (*n* = 3). Data are represented as mean ± SD. *p* values were calculated using a two-tailed unpaired Student’s t test. ∗ indicates *p* <0.05, ∗∗ indicates *p* <0.01, ∗∗∗ indicates *p* <0.001.

We first assessed the cytotoxicity of eupalinolide A (EA), EB, and eupalinolide O (EO), extracted from *Eupatorium lindleyanum*, on the pancreatic cancer cell line MiaPaCa-2. Using the CCK8 assay, we observed a significant reduction in MiaPaCa-2 cell viability following drug treatment, with EB having the most pronounced effect (Figure 1A). Additionally, when comparing the effects on three pancreatic cancer cell lines (MiaPaCa-2, PANC-1, and PL-45) to normal pancreatic cells (HPNE) after drug treatment, EB demonstrated a significantly stronger effect on pancreatic cancer cells than on normal cells (Figure 1B). To further validate our findings, we included oxaliplatin as a positive control in our functional assays. Oxaliplatin is a first-line chemotherapeutic agent for pancreatic cancer, with established efficacy against pancreatic cancer cells, thus serving as a benchmark for comparison. Edu fluorescence staining demonstrated a reduction in Edu fluorescence intensity in the EB treatment group compared to the control, suggesting a decreased rate of DNA synthesis subsequent to drug treatment (Figures 1C and 1D). Moreover, the colony formation assay reflected that EB treatment notably reduced the number and size of colonies of pancreatic cancer cells, further confirming the inhibitory effect of EB on the proliferative capacity of these cells, with effects comparable to those observed with oxaliplatin (Figure 1E). The wound healing assay revealed that EB treatment delayed the ability of pancreatic cancer cells to migrate to the scratched area, indicating reduced migratory capacity, similar to the results seen with oxaliplatin treatment (Figures 1F and 1G). Transwell assay results demonstrated a significant decrease in the ability of EB-treated pancreatic cancer cells to penetrate the matrix gel and migrate to the lower layer, suggesting that EB has a clear inhibitory effect on the invasive ability of pancreatic cancer cells, paralleling the inhibitory effects observed with oxaliplatin (Figure 1H).

The aforementioned results indicate that EB can inhibit the biological functions of pancreatic cancer cells. EB’s suppression of the malignant biological characteristics of pancreatic cancer cells across multiple dimensions demonstrates its potential anticancer activity, which is further supported by its comparable efficacy to oxaliplatin.

### EB exhibits therapeutic effects in xenografted tumor-bearing nude mice

**Figure 2:**
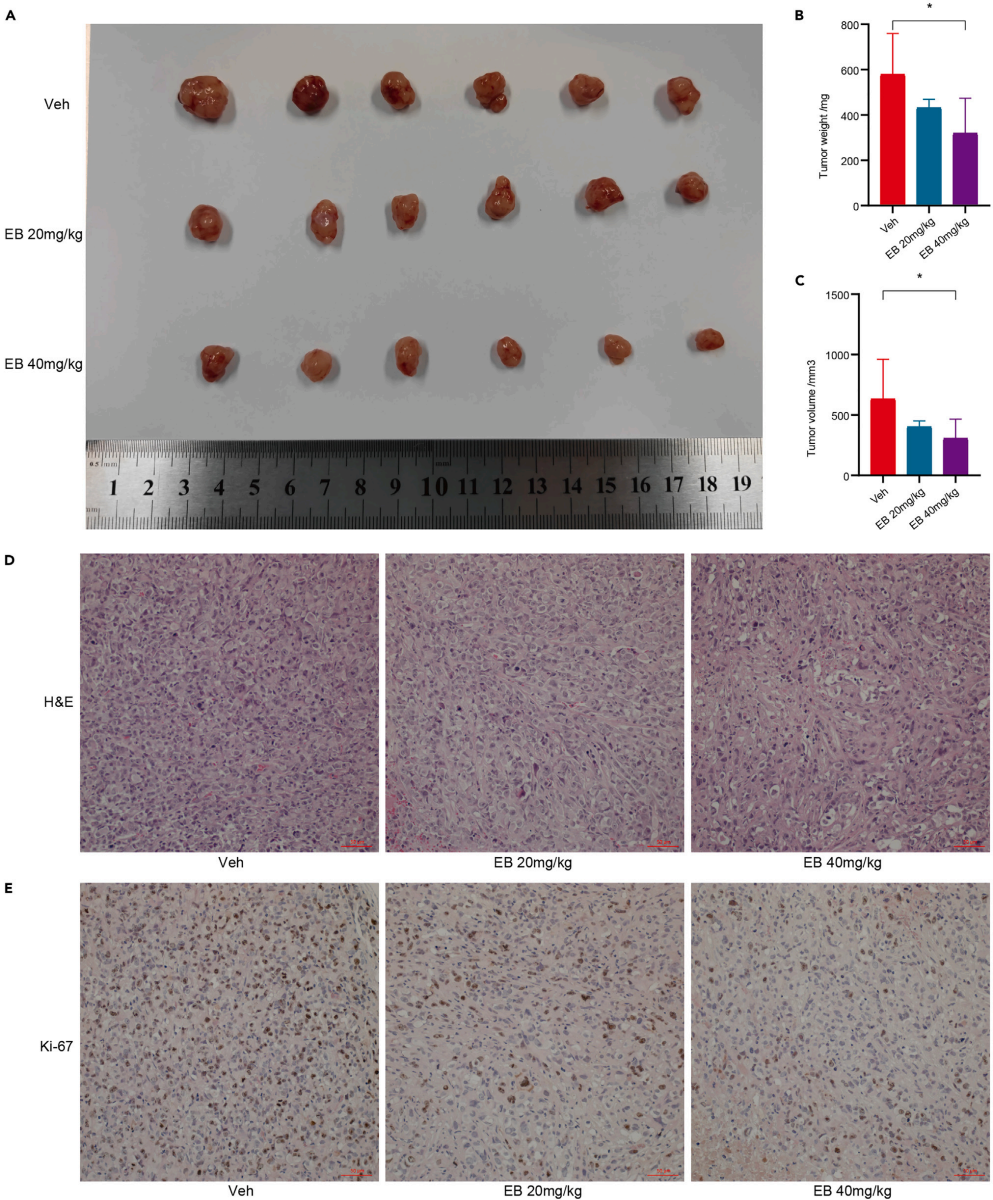
EB exhibits therapeutic effects in xenografted tumor-bearing nude mice (A) Tumor progression in nude mice following intraperitoneal injection of varying doses of EB. (B) Statistical chart of tumor weights in nude mice. (C) Statistical chart of tumor volumes in nude mice. (D) Hematoxylin and eosin (H&E) staining of tumor tissues. (E) Immunohistochemical staining for Ki-67 in tumor tissues. Six replicates per group were set up (*n* = 6). Data are represented as mean ± SD. *p* values were calculated using a two-tailed unpaired Student’s t test. ∗ indicates *p* value <0.05.

In our study, we established a xenograft tumor model by implanting PANC-1 pancreatic cancer cells into nude mice. Tumor growth in mice treated with EB was significantly slower (Figure 2A), and both tumor volume and weight were substantially reduced compared to the control group (Figures 2B and 2C). Hematoxylin and eosin (H&E) staining was conducted on mouse tissues to delineate the pancreatic cancer tissue (Figure 2D). Immunohistochemical analysis revealed a decrease in Ki-67 expression (Figure 2E), indicating that EB may exert its antitumor effects by inhibiting cell proliferation.

### Analysis of potential mechanisms for EB controlling pancreatic cancer cell death

**Figure 3:**
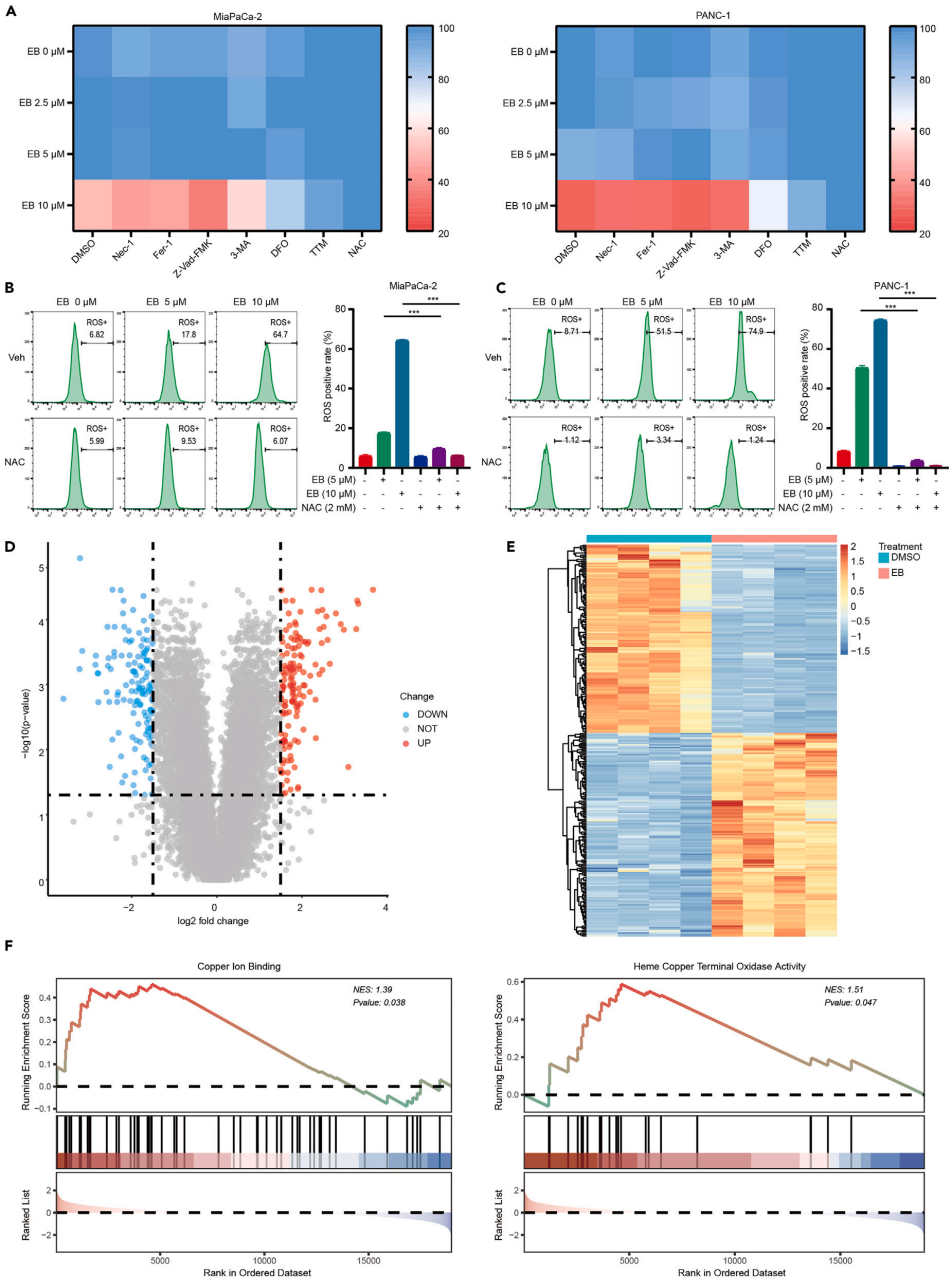
Analysis of potential mechanisms for EB controlling pancreatic cancer cell death (A) Impact on cell viability of MiaPaCa-2 (left) and PANC-1 (right) upon treatment with inhibitors: Nec-1 (necroptosis), Fer-1 (ferroptosis), Z-Vad-FMK (apoptosis), 3-MA (autophagy), DFO (iron chelation), TTM (copper chelation), and NAC (antioxidant) following EB exposure. (B) Left: flow cytometry detection of ROS in MiaPaCa-2 cells treated with EB. Right: the statistical chart of ROS positive rate. (C) Left: flow cytometry detection of ROS in PANC-1 cells treated with EB. Right: the statistical chart of ROS positive rate. (D) Volcano plots of gene expression in PANC-1 cells with and without EB treatment. (E) Heatmaps of differentially expressed genes in PANC-1 cells with and without EB treatment. (F) GSEA analysis of RNA-seq data for PANC-1 cells treated with and without EB. Three replicates per group were set up (*n* = 3). Data are represented as mean ± SD. *p* values were calculated using a two-tailed unpaired Student’s t test. ∗ indicates *p* value <0.05, ∗∗ indicates *p* value <0.01, ∗∗∗ indicates *p* value <0.001.

In experiments examining the cell death mechanism of EB in pancreatic cancer cells, various inhibitors were used to reveal potential death modes. Employing inhibitors including Nec-1 (necroptosis inhibitor), Fer-1 (ferroptosis inhibitor), Z-Vad-FMK (apoptosis inhibitor), 3-MA (autophagy inhibitor), DFO (iron ions chelator), tetrathiomolybdate (TTM) (copper ions chelator), and NAC (antioxidant), and monitoring cell viability with the CCK-8 assay, we found that TTM and NAC could significantly reverse the cell death in pancreatic cancer cells caused by EB (Figure 3A). To confirm the role of ROS, we performed flow cytometry to measure the levels of ROS in pancreatic cancer cells after EB treatment. The results showed a significant increase in ROS levels in EB-treated cells, which was effectively suppressed by NAC (Figures 3B and 3C). This finding was further supported by fluorescent imaging, which confirmed elevated ROS levels in the EB treatment group and their reduction upon NAC addition (Figure S1A).

To elucidate the molecular mechanism of EB, we utilized RNA sequencing (RNA-seq) technology to analyze the differential gene expression in PANC-1 cells before and after treatment with 10 μM EB. Differential expression analysis was performed using the limma R package on RNA-seq data. By setting a threshold of *p* < 0.05 and logFC > 1.5, we identified significantly differentially expressed genes, which were visualized in a volcano plot (Figure 3D) and a heatmap (Figure 3E). To further investigate the pathways involved, we utilized the R package clusterProfiler to perform Kyoto Encyclopedia of Genes and Genomes (KEGG) enrichment analysis on the differentially expressed genes, comprising 145 upregulated and 134 downregulated genes. The analysis revealed significant enrichment in several pathways for the upregulated genes, with the mitogen-activated protein kinase (MAPK) pathway being particularly prominent (Figures S1B and S1C), suggesting it as a potential key pathway in EB’s anti-pancreatic cancer activity. Western blot analysis revealed no significant changes in the phosphorylation levels of extracellular regulated protein kinases 1/2 (ERK1/2) and p38 MAPK in PANC-1 and MiaPaCa-2 cells post EB treatment. However, a marked increase was noted in the phosphorylation levels of c-Jun N-terminal kinase (JNK) (Figure S1D), indicating that EB may exert its anticancer effects through JNK pathway activation. Further exploration of the role of JNK activity in EB-induced cell death using the specific inhibitor SP600125 demonstrated that while it effectively inhibited JNK phosphorylation (Figure S1E), it did not mitigate EB-induced cell death (Figure S1F), suggesting that the cell death induced by EB does not entirely depend on the activation of the JNK signaling pathway. Therefore, although the increase in JNK phosphorylation is significant in EB-treated pancreatic cancer cells, other parallel pathways or factors must be involved in EB’s cytotoxic effects. In the process of validating KEGG signaling pathways, we also investigated proteins related to ferroptosis, which was notably enriched (Figure S1B), such as glutathione peroxidase 4 (GPX4) and acyl-CoA synthetase long-chain family member 4 (ACSL4), and no significant alterations were observed following EB treatment (Figure S1G). When examining the expression of apoptosis-related proteins, we found that EB induces cleavage of caspase 3, caspase 9, and poly ADP-ribose polymerase (PARP) (Figure S2A). These findings were further validated using apoptosis flow cytometry (Figure S2B) and fluorescence imaging (Figure S2C), both of which confirmed the presence of apoptosis in EB-treated cells. However, the apoptosis inhibitor Z-Vad-FMK failed to reverse EB-induced cell death (Figure 3A), suggesting that while EB induces apoptotic pathways, other mechanisms might also be contributing to cell death.

Given the observed ineffectiveness of MAPK pathway inhibition and the known relationship between copper ions and cellular processes, we performed gene set enrichment analysis (GSEA). The analysis revealed significant enrichment in pathways related to copper ion binding and heme copper terminal oxidase activity (Figure 3F), both of which are closely associated with copper metabolism.

Based on these findings, we focused our subsequent research on the role of copper in EB-induced cell death. Our study suggests that EB promotes ROS generation and interacts with copper ion pathways, leading to cuproptosis in pancreatic cancer cells. This insight provides a deeper understanding of the mechanisms by which EB exerts its anticancer effects and highlights the potential of targeting copper metabolism in pancreatic cancer therapy.

### EB increases intracellular copper levels and modulates cuproptosis-related proteins

**Figure 4:**
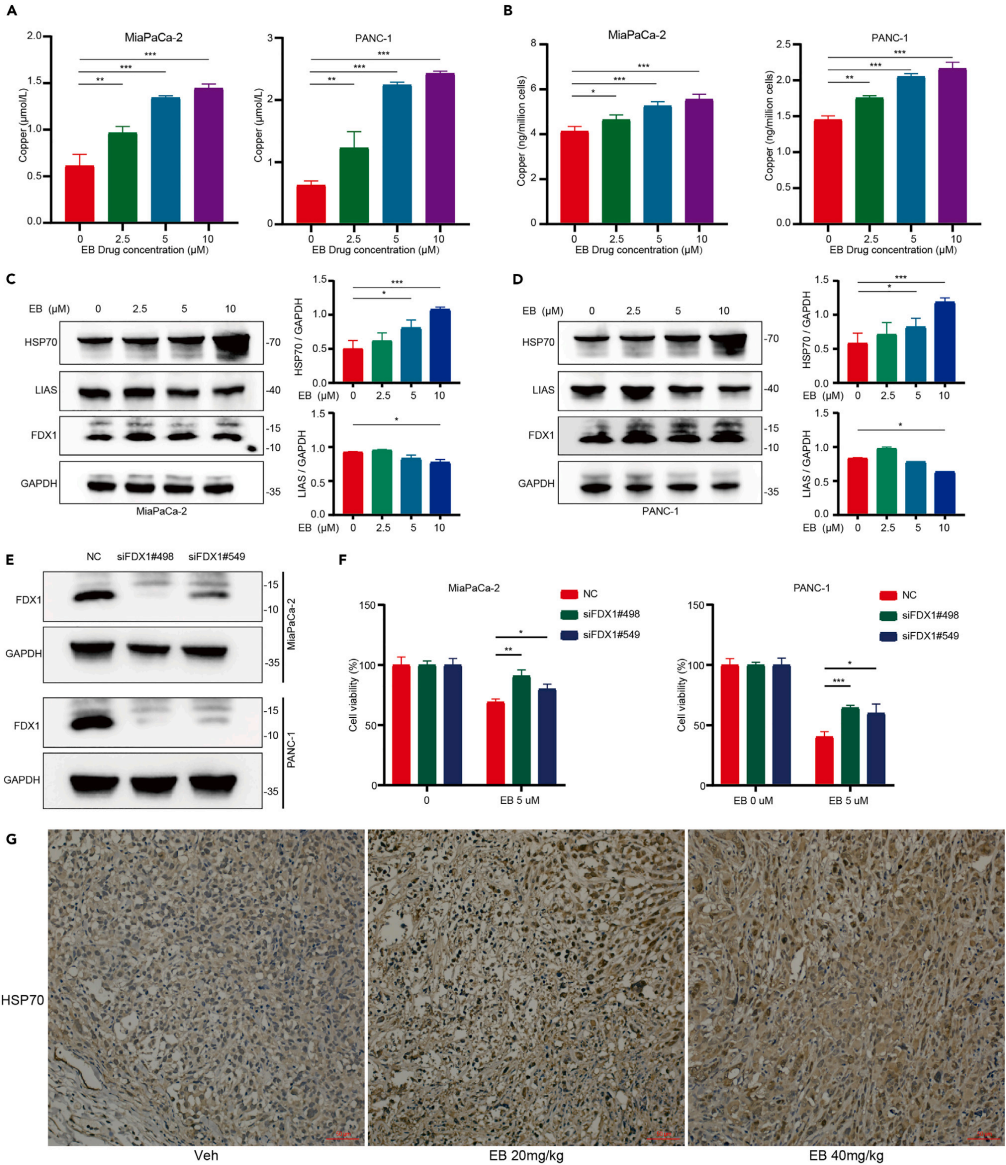
EB increases intracellular copper levels and modulates cuproptosis-related proteins (A) Intracellular copper ion levels after treatment with varying concentrations of EB. (B) Utilization of ICP-MS for the determination of cellular copper ion concentrations under varying EB concentration gradients. (C) Left: expression of cuproptosis related proteins in MiaPaCa-2 under EB concentration gradients. Right: quantitative analysis of protein bands. (D) Left: expression of cuproptosis related proteins in PANC-1 under EB concentration gradients. Right: quantitative analysis of protein bands. (E) FDX1 protein expression after knockdown in pancreatic cancer cells. (F) Impact on cell viability after EB treatment post-FDX1 knockdown. (G) Immunohistochemical staining for HSP70 in nude mouse xenograft tumor tissues. Three replicates per group were set up (*n* = 3). Data are represented as mean ± SD. *p* values were calculated using a two-tailed unpaired Student’s t test. ∗ indicates *p* value <0.05, ∗∗ indicates *p* value <0.01, ∗∗∗ indicates *p* value <0.001.

To investigate the impact of EB on intracellular copper ion levels in pancreatic cancer cells, we employed the complexing method to measure copper content following EB treatment. Our results indicated a significant increase in intracellular copper ion levels after EB treatment (Figure 4A). This finding was further validated using inductively coupled plasma mass spectrometry (ICP-MS), which confirmed the elevated copper levels in EB-treated cells (Figure 4B). Additionally, we utilized a copper ion probe to visualize and quantify the intracellular copper ion levels. The probe method corroborated our previous results, showing a marked increase in copper ion levels within cells treated with EB (Figures S3A and S3B).

To explore the mechanisms underlying EB-induced cuproptosis, we examined the expression levels of three key proteins associated with cuproptosis using western blot analysis. The results revealed that EB treatment significantly increased the protein levels of HSP70 and decreased the protein levels of lipoic acid synthetase (LIAS). However, the impact on FDX1 protein levels was not significant (Figures 4C and 4D). Given the minor effect of EB on FDX1, we performed FDX1 knockdown experiments (Figure 4E) to further investigate its role. Knockdown of FDX1 effectively reversed the cell death induced by EB, suggesting that FDX1 is critical for EB-mediated cytotoxicity (Figure 4F). Furthermore, we conducted immunohistochemistry (IHC) on tumor tissues from nude mice previously treated with EB. The IHC results demonstrated an increase in HSP70 protein levels in the EB-treated group, consistent with our *in vitro* findings (Figure 4G).

These results collectively indicate that EB can elevate intracellular copper levels and modulate the expression of key cuproptosis-related proteins, particularly HSP70 and LIAS. The reversal of EB-induced cell death by FDX1 knockdown further underscores the importance of copper ion regulation and cuproptosis in the anticancer activity of EB.

### Copper ions modulation affects EB-induced cell death

**Figure 5:**
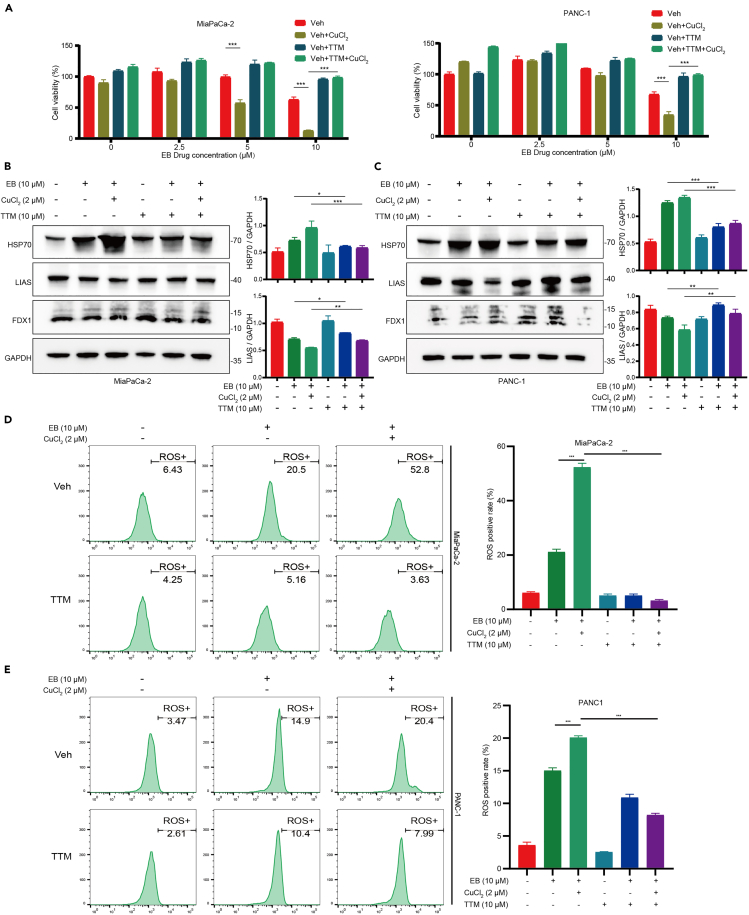
Copper ions modulation affects EB-induced cell death (A) Effect of 2 μM CuCl_2_ and 10 μM TTM on EB-mediated cell viability in MiaPaCa-2 (left) and PANC-1 (right). (B) Left: impact of 2 μM CuCl_2_ and 10 μM TTM on EB-mediated regulation of copper-induced cell death-related proteins in MiaPaCa-2. Right: quantitative analysis of protein bands. (C) Left: impact of 2 μM CuCl_2_ and 10 μM TTM on EB-mediated regulation of copper-induced cell death-related proteins in PANC-1. Right: quantitative analysis of protein bands. (D) Left: flow cytometric analysis of ROS in MiaPaCa-2 cells treated with EB under CuCl_2_ and TTM intervention. Right: The statistical chart of ROS positive rate. (E) Left: flow cytometric analysis of ROS in PANC-1 cells treated with EB under CuCl_2_ and TTM intervention. Right: the statistical chart of ROS positive rate. Three replicates per group were set up (*n* = 3). Data are represented as mean ± SD. *p* values were calculated using a two-tailed unpaired Student’s t test. ∗ indicates *p* value <0.05, ∗∗ indicates *p* value <0.01, ∗∗∗ indicates *p* value <0.001.

To further investigate the role of copper ions in EB-induced cell death, we used the CCK8 assay to assess the effects of CuCl_2_ and TTM on pancreatic cancer cells treated with EB. Our results showed that the addition of CuCl_2_ exacerbated EB-induced cell death, while TTM effectively reversed this effect (Figure 5A). We also examined the impact of CuCl_2_ and TTM on the expression levels of cuproptosis-related proteins in EB-treated cells. Western blot analysis revealed that CuCl_2_ increased the levels of HSP70 protein and decreased the levels of LIAS protein in cells treated with EB. These changes were effectively reversed by TTM (Figures 5B and 5C). Furthermore, we used flow cytometry to measure the levels of ROS in pancreatic cancer cells treated with EB in the presence of CuCl_2_ and TTM. The results indicated that CuCl_2_ enhanced EB-induced ROS levels, which could be reversed by TTM (Figures 5D and 5E).

These findings suggest that copper ions play a significant role in modulating the effects of EB on pancreatic cancer cells. The enhancement of EB-induced cell death and changes in protein expression by CuCl_2_, along with the reversal of these effects by TTM, highlight the critical involvement of copper ion regulation and ROS generation in EB-mediated anticancer activity.

### EB enhances ES-induced cell death in pancreatic cancer cells

**Figure 6:**
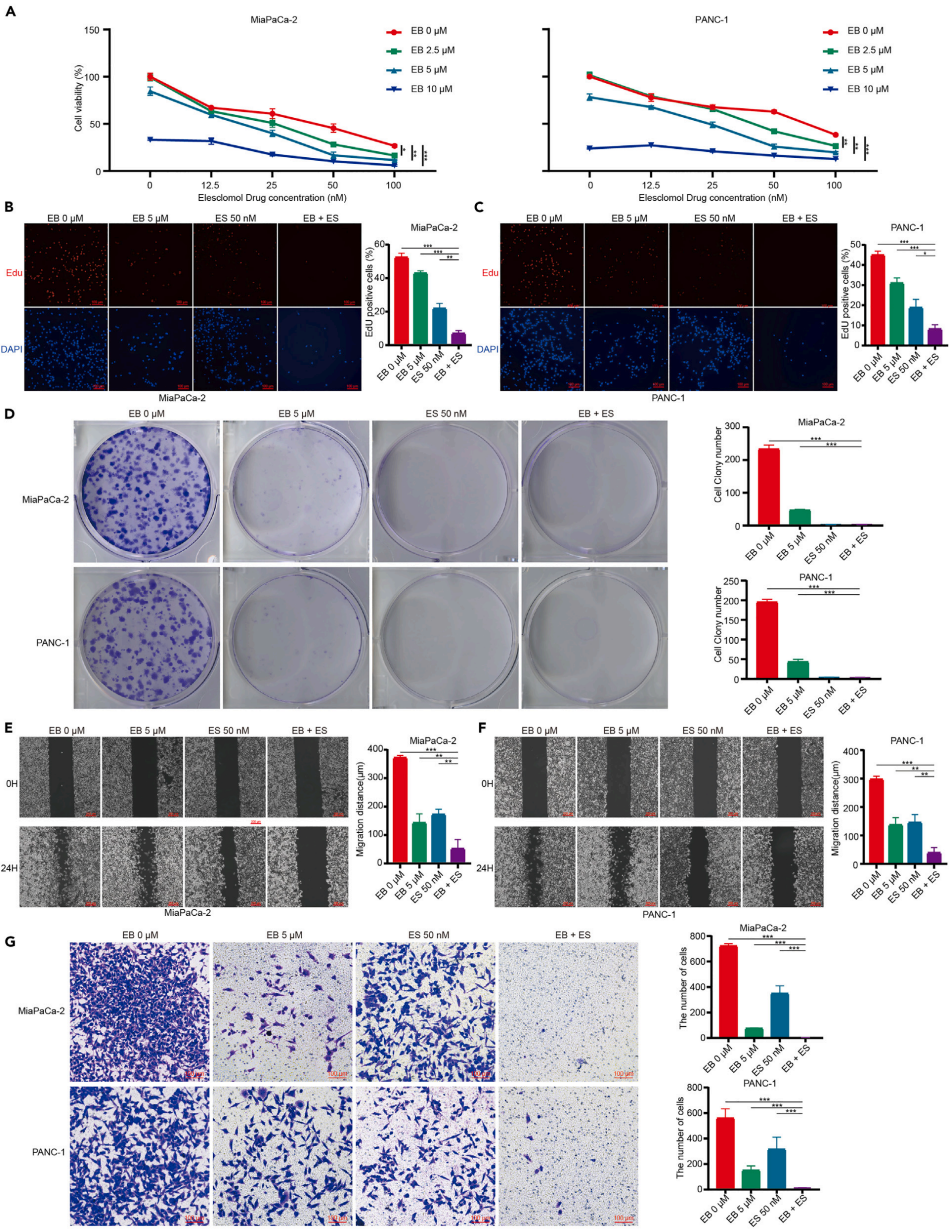
EB enhances ES-induced cell death in pancreatic cancer cells (A) Effects on cell viability of MiaPaCa-2 (left) and PANC-1 (right) with combined EB and ES treatment under 2 μM CuCl_2_. (B) Left: Edu fluorescence staining of MiaPaCa-2 cells after treatment with control, 5 μM EB, 50 nM ES, or the combination of EB and ES. Right: statistical chart of the percentage of Edu-positive cells. (C) Left: Edu fluorescence staining of PANC-1 cells post-treatment with control, 5 μM EB, 50 nM ES, or the combination of EB and ES. Right: statistical chart of the percentage of Edu-positive cells. (D) Left: colony formation assay of pancreatic cancer cells after treatment with control, 5 μM EB, 50 nM ES, or the combination of EB and ES. Right: statistical chart of the number of colonies formed. (E) Left: wound healing assay of MiaPaCa-2 cells treated with control, 5 μM EB, 50 nM ES, or the combination of EB and ES. Right: statistical chart of the wound closure distance. (F) Left: wound healing assay of PANC-1 cells treated with control, 5 μM EB, 50 nM ES, or the combination of EB and ES. Right: statistical chart of the wound closure distance. (G) Left: invasion assay of pancreatic cancer cells after treatment with control, 5 μM EB, 50 nM ES, or the combination of EB and ES. Right: associated statistical chart. Three replicates per group were set up (*n* = 3). Data are represented as mean ± SD. *p* values were calculated using a two-tailed unpaired Student’s t test. ∗ indicates *p* value <0.05, ∗∗ indicates *p* value <0.01, ∗∗∗ indicates *p* value <0.001.

ES is a known cuproptosis inducer. To evaluate the synergistic effects of EB and ES on pancreatic cancer cell viability, we conducted CCK8 assays. Our results demonstrated that EB significantly enhanced ES-induced cell death (Figure 6A). For subsequent biological function assays, we selected 5 μM of EB and 50 nM of ES for combined treatment, based on their individual and combined efficacy. We assessed the combined effects of EB and ES on various biological functions of pancreatic cancer cells. The Edu fluorescence staining showed a significant reduction in Edu fluorescence intensity in the combined treatment group compared to either EB or ES alone, indicating a further decreased rate of DNA synthesis (Figures 6B and 6C). The colony formation assay revealed a more pronounced reduction in the number and size of colonies in the combined treatment group, confirming the enhanced inhibitory effect on cell proliferation (Figure 6D). The wound healing assay demonstrated that the combined treatment significantly delayed the migration of pancreatic cancer cells to the scratched area, indicating a reduced migratory capacity (Figures 6E and 6F). Additionally, the transwell assay results showed a substantial decrease in the ability of pancreatic cancer cells to penetrate the matrix gel and migrate to the lower layer under the combined treatment, further suggesting a synergistic inhibitory effect on cell invasion (Figure 6G).

These results indicate that EB enhances the anticancer effects of ES, significantly amplifying the inhibition of multiple malignant biological characteristics of pancreatic cancer cells. This synergistic interaction underscores the potential of combined EB and ES treatment as a promising therapeutic strategy for pancreatic cancer.

### Copper modulation affects the synergistic effects of EB and ES in pancreatic cancer cells

**Figure 7:**
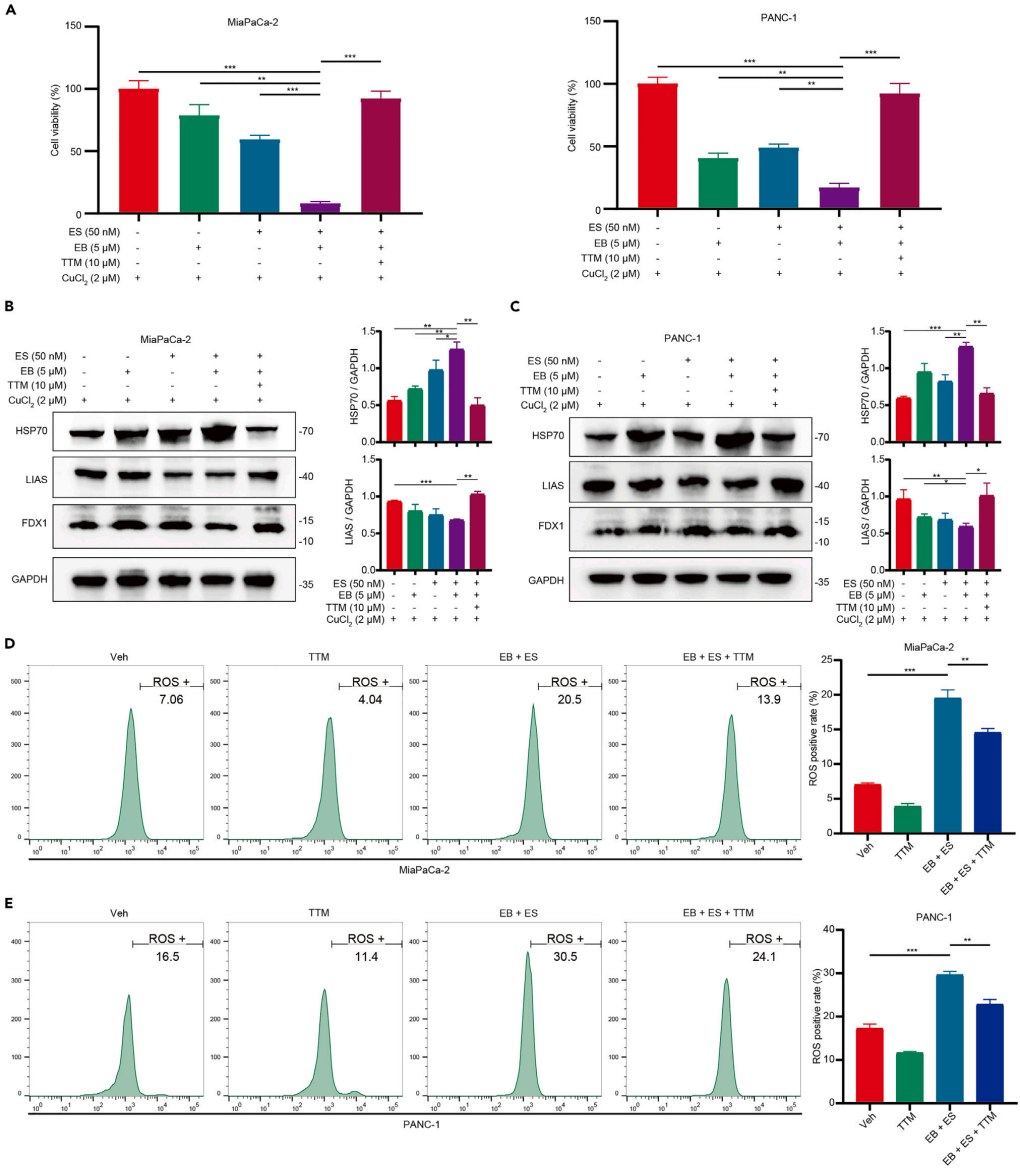
Copper modulation affects the synergistic effects of EB and ES in pancreatic cancer cells (A) Inhibitory effects of 10 μM TTM on the synergistic action of EB and ES under 2 μM CuCl_2_. (B) Left: alterations in copper-induced death proteins following inhibition by TTM of the combined action of EB and ES under 2 μM CuCl_2_ in MiaPaCa-2. Right: quantitative analysis of protein bands. (C) Left: alterations in copper-induced death proteins following inhibition by TTM of the combined action of EB and ES under 2 μM CuCl_2_ in PANC-1. Right: quantitative analysis of protein bands. (D) Left: Flow cytometry analysis of the reversal effect of TTM on ROS levels in cells treated with EB and ES under 2 μM CuCl_2_ in MiaPaCa-2. Right: the statistical chart of ROS positive rate. (E) Left: Flow cytometry analysis of the reversal effect of TTM on ROS levels in cells treated with EB and ES under 2 μM CuCl_2_ in PANC-1. Right: the statistical chart of ROS positive rate. Three replicates per group were set up (*n* = 3). Data are represented as mean ± SD. *p* values were calculated using a two-tailed unpaired Student’s t test. ∗ indicates *p* value <0.05, ∗∗ indicates *p* value <0.01, ∗∗∗ indicates *p* value <0.001.

To further explore the role of copper ions in the combined effects of EB and ES on pancreatic cancer cells, we conducted CCK8 assays to assess the impact of CuCl_2_ and TTM on cell viability. Our results indicated that CuCl_2_ enhanced the cytotoxic effects of the EB and ES combination, whereas TTM effectively suppressed this enhancement (Figure 7A). We also examined the influence of CuCl_2_ and TTM on the expression of key cuproptosis-related proteins under the combined treatment of EB and ES. Western blot analysis revealed that CuCl_2_ increased the protein levels of HSP70 and decreased the protein levels of LIAS in the combined treatment group. These effects were reversed by TTM (Figures 7B and 7C). Furthermore, flow cytometry was used to measure the levels of ROS in pancreatic cancer cells under the combined treatment of EB and ES, with and without CuCl_2_ and TTM. The results showed that the combined treatment significantly elevated ROS levels, which were further enhanced by CuCl_2_ and partially suppressed by TTM (Figures 7D and 7E).

These findings suggest that copper ions play a critical role in modulating the synergistic effects of EB and ES on pancreatic cancer cells. The enhancement of combined treatment-induced cell death, changes in protein expression, and ROS levels by CuCl_2_, along with the suppression of these effects by TTM, highlight the importance of copper ion regulation in the therapeutic efficacy of EB and ES.

## Discussion

This study discovered that the drug EB exhibits significant inhibitory effects on the pancreatic cancer cell lines PANC-1 and MiaPaCa-2, showing more marked cytotoxicity against these cancer cells than against normal pancreatic cells. A substantial challenge in clinical pancreatic cancer treatment is the lack of specificity, which often results in significant side effects. The findings suggest potential for EB as a therapeutic agent targeting pancreatic cancer.

EB demonstrated anticancer activity *in vitro* by inhibiting the proliferation, colony formation, and migration of pancreatic cancer cells, suggesting that its effect might result from inhibition of cell viability and promotion of cell dead. Reduction in Edu fluorescence staining directly indicated an inhibitory effect of EB on DNA synthesis, while scratch and transwell assays implied suppression of pancreatic cancer cell migration and invasion. These findings are consistent with the aggressive nature of pancreatic cancer and its contribution to poor prognosis. The therapeutic efficacy of EB was further confirmed *in vivo* by the slowed growth of tumors in a nude mouse model, and decreased expression of Ki-67 highlighted a strong suppressive effect on tumor cell proliferation. These findings confirm the potential clinical applicability of EB and lay the foundation for subsequent pharmacological and toxicological evaluations.

Molecularly, the action of EB involves modulation of the MAPK pathway, particularly the activation of JNK isoforms. Considering the extensive research on the role of the JNK pathway in cancer in regulating various cellular responses, including stress and cell fate decisions,^19^^,^^20^ the unchanged levels of ERK and p38 MAPK suggest specificity or selectivity in the action of EB. Notably, despite JNK activation, inhibition of the JNK pathway did not reduce the cell death induced by EB, indicating that its antitumor effects might be mediated through more complex mechanisms than just the JNK pathway. Furthermore, the inconspicuous changes in ferroptosis-related pathway proteins suggest molecular mechanisms underpinning the effects of EB. These findings suggest that effective pancreatic cancer treatment may require targeting multiple signaling pathways and highlight the mechanism of EB as a valuable direction for future research.

As an essential trace nutrient for humans, copper metabolism is finely regulated by evolutionarily conserved homeostatic mechanisms. Copper toxicity occurs when its concentration exceeds a certain threshold, a principle utilized in the development of copper ionophores such as ES for anticancer therapy. Though copper is physiologically essential, excess copper can generate ROS through Fenton or Fenton-like reactions, leading to cytotoxicity.^21^^,^^22^ Copper can facilitate tumor development by promoting angiogenesis, proliferation, and metastasis.^23^^,^^24^ The relationship between EB and copper ions introduces an aspect to understanding the anticancer mechanism of EB. Our findings suggest that EB disrupts metal ion homeostasis in pancreatic cancer cells, resulting in cytotoxicity. Post-EB treatment, a significant increase in intracellular copper levels was observed, prompting intriguing questions about the drug’s mechanism of action. EB enhances copper influx and hampers efflux via transport proteins (ATP7A/7B),^25^ or interaction with copper-binding proteins needs clarification. Results from this study also strongly suggest that ROS production and copper ion metabolism play pivotal roles in pancreatic cancer cell death induced by EB. The dual modulation of ROS and copper ions has been identified as a significant cell death signal in cancer research, with the protective effects of TTM and NAC lending further support. These findings not only underscore the importance of the copper-dependent cell death pathway in the anticancer effects mediated by EB but also highlight the potential cuproptosis and antioxidant defense mechanisms as targets in pancreatic cancer therapy.

Recent studies on cuproptosis revealed that cuproptosis primarily affects cells relying on respiration and the Tricarboxylic Acid (TCA) cycle. It involves copper binding to fatty acyl groups, causing a cascade of events: aggregation of fatty acylated proteins, depletion of iron-sulfur cluster proteins, activation of HSP70, oxidative stress induction, and culminating in cell death.^15^ Prior studies showed that EB raises HSP70 levels,^26^^,^^27^ but did not link this to copper ions or copper-triggered cell death. Herein, we reaffirm that EB can facilitate the upregulation of HSP70. Our experimental results imply that EB potentially affects copper ions, triggering cuproptosis, characterized by increased HSP70 and decreased LIAS levels, which may be associated with the regulation of protein lipoylation in the copper death process. HSP70 is a type of heat shock protein that plays a vital role in the cellular stress response.^15^ The rise in HSP70 levels might be a protective mechanism for cells to counter the stress response induced by EB. Specifically, HSP70 helps in the correct folding of proteins, preventing improper folding and aggregation, thereby maintaining the protein homeostasis within the cell. On the other hand, LIAS is one of the key enzymes during the copper death process, involved in the lipoylation of proteins such as dihydrolipoyl transacetylase (DLAT), which is a subunit of the E2 enzyme complex within the pyruvate dehydrogenase complex. The lipoylation state of DLAT is a significant marker of copper-induced death.^15^ The reduction of LIAS levels by EB may disrupt the copper death process by inhibiting the lipoylation of DLAT. This change could lead to a disturbance in the normal metabolic regulation of copper ions within the cell, accumulating to toxic levels and ultimately triggering cell death. In conclusion, the increase in HSP70 and the decrease in LIAS levels respectively reflect the cellular stress response to EB and the initiation of copper-induced death. HSP70 might protect cells by sustaining protein homeostasis, while the reduction in LIAS could facilitate copper death by inhibiting the lipoylation of DLAT. Future research should delve into the specific molecular mechanisms of these proteins in the context of copper-induced death and confirm their crucial roles during the copper death process induced by the drug EB.

The observed modulation of HSP70 and LIAS protein levels by EB and the reversal of these changes upon copper chelation by TTM implicate these proteins as potential mediators of cuproptosis. Knocking down FDX1, a crucial protein in cuproptosis,^15^^,^^22^ was found to partially reverse the cell death induced by EB in this study, indirectly confirming that EB promotes pancreatic cancer cell death by triggering cuproptosis. The contribution of FDX1 to EB’s mechanism indicates a complex interaction between copper homeostasis and cuproptosis. Iron (Fe) and copper (Cu) ions are key drivers of regulated cell death (RCD), inducing ferroptosis and cuproptosis respectively, and leading to cell damage and various cell deaths.^28^ Studies on cell death modes revealed that DFO, a ferroptosis inhibitor, could also suppress EB effects (Figure 3A). However, subsequent validations did not detect changes in the critical ferroptosis protein GPX4, suggesting the need for further research into EB’s specific protein modulation within the ferroptosis pathway.

ES, as a potent copper ionophore, also serves as an anti-cancer agent targeting mitochondrial metabolism. Formerly considered an inducer of oxidative stress,^29^ it has also been found to inhibit cancer by inducing copper-dependent apoptosis.^30^ The combination of EB and ES leads to synergistic effects, markedly enhancing the cytotoxicity in pancreatic cancer cells. Notably, the presence of TTM abrogates the lethal synergy between EB and copper ions. This protective role of TTM underscores the crucial involvement of copper in EB-induced cell death. These results suggest that EB does not act in isolation but can be part of a larger network of interactions involving copper metabolism.

Furthermore, the increase in ROS levels upon treatment with EB and ES, and its subsequent reduction by copper chelation, posits oxidative stress as a mediator of cell death. However, several questions remain; the specific copper-binding entities within the cell affected by EB, the direct interaction of EB with these molecules, and the downstream effects warrant further investigation. Additionally, the therapeutic window for EB’s anticancer activity relative to copper toxicity needs careful delineation, as systemic copper accumulation can have deleterious effects.

The study suggests that EB-induced copper and ROS accumulation sensitize cancer cells to ES, enhancing synergistic effects and offering a potential strategy for combination therapies targeting metal ion dysregulation in cancer treatment. Future research should aim to characterize copper-binding sites or transport proteins affected by EB and explore the therapeutic potential of this drug in subsequent pancreatic cancer treatments.

### Limitations of the study

While our study provides promising insights into the anticancer effects of EB in pancreatic cancer, several limitations must be acknowledged. Firstly, the exact molecular mechanisms underlying EB-induced cuproptosis remain to be fully elucidated, particularly the specific roles of copper-binding proteins and mitochondrial dynamics. Additionally, our *in vitro* findings may not fully replicate the complex tumor microenvironment present *in vivo*, which could influence the efficacy and safety of EB. Moreover, while we demonstrated synergistic effects with ES, the long-term effects and potential toxicity of this combination therapy in clinical settings require further investigation. Finally, our study relied on xenograft mouse models, which, while informative, may not completely capture the human disease pathology. Future studies should aim to validate these findings in more clinically relevant models and explore potential side effects in greater detail.

## STAR★Methods

### Key resources table

**Table:** 

REAGENT or RESOURCE	SOURCE	IDENTIFIER
**Antibodies**		

LIAS Polyclonal antibody	proteintech	CAT#11577-1-AP; RRID: AB_2135972
HSP70 Polyclonal antibody	proteintech	CAT#10995-1-AP; RRID: AB_2264230
FDX1 Polyclonal antibody	proteintech	CAT#12592-1-AP; RRID: AB_11182486
Phospho-MAPK Family Antibody Sampler Kit	CST	CAT#9910T
MAPK Family Antibody Sampler Kit	CST	CAT#9926T
GAPDH	CST	CAT#5174; RRID: AB_10622025
HRP-conjugated Affinipure Goat Anti-Rabbit IgG(H+L)	proteintech	CAT#SA00001-2; RRID: AB_2722564
Ki-67	ZSGB-BIO	CAT#ZM-0166; RRID: AB_2890067
C3	CST	CAT#14220; RRID: AB_2798429
CC3	CST	CAT#9661; RRID: AB_2341188
C9	CST	CAT#9508; RRID: AB_2068620
CC9	CST	CAT#20750; RRID: AB_2798848
PARP	CST	CAT#9542; RRID: AB_2160739
GPX4	Abcam	CAT#ab125066; RRID: AB_10973901
ACSL4	proteintech	CAT#22401-1-AP; RRID: AB_2832995
β-tubulin	CST	CAT#2146; RRID: AB_2210545

**Chemicals, peptides, and recombinant proteins**		

DMEM	Gibco	CAT#C11995500B
Fetal Bovine Serum (FBS)	ExCell Bio	CAT#FSP500
Trypsin	Gibco	CAT#25200072
PBS	Servicebio	CAT#G4250
Eupalinolide A	ChemFaces	CAT#CFN90381
Eupalinolide O	ChemFaces	CAT#CFN91839
Eupalinolide B	AbMole	CAT#M4619
Elesclomol	Selleck	CAT#S1052
Z-VAD-FMK	Selleck	CAT#S7023
SP600125	Selleck	CAT#S1460
Deferoxamine mesylate (DFO)	Selleck	CAT#S5742
3-Methyladenine (3-MA)	Selleck	CAT#S2767
Necrostatin-1	Selleck	CAT#S8037
Ferrostatin-1 (Fer-1)	Selleck	CAT#S7243
Oxaliplatin	Selleck	CAT#S1224
N-Acetylcysteine	MedChemExpress	CAT#HY-B0215
Tetrathiomolybdate	MedChemExpress	CAT#HY-W076067
Coppersensor 1	MedChemExpress	CAT#HY-141511
Lipo3000	Invitrogen	CAT#L3000015
DMSO	MP Biomedicals	CAT#196055

**Critical commercial assays**		

Cell Copper Colorimetric Assay Kit	Elabscience	CAT#E-BC-K775-M
The Cell-Light EdU Apollo567 *In Vitro* Kit	Ribo Bio	CAT#C10310-1
Cell counting kit-8 (CCK8)	NCM Biotech	CAT#C6005
FITC Annexin V detection kit	KEYGEN BIOTECH	CAT#KGA108
Reactive Oxygen Species Assay Kit, H2DCFDA	KEYGEN BIOTECH	CAT#KGA7501

**Deposited data**		

Raw and analyzed data	This paper	GSE256455

**Experimental models: Cell lines**		

PANC-1	ATCC	N/A
MiaPaCa-2	ATCC	N/A
PL-45	ATCC	N/A
HPNE	ATCC	N/A

**Experimental models: Organisms/strains**		

Balb/c nude mice	Gempharmatech	N/A

**Oligonucleotides**		

FDX1-Homo-498 CCUGGCUUGUUCAACCUGUTT ACAGGUUGAACAAGCCAGGTT FDX1-Homo-549 GUUAGAUGCAAUCACUGAUTT AUCAGUGAUUGCAUCUAACTT	Genepharma	N/A

**Software and algorithms**		

GraphPad Prism 8 software	GraphPad Software Inc	https://www.graphpad.com/
ImageJ software	US National Institutes of Health	https://imagej.net/
R version 4.2.1	The R Foundation	http://r-project.org
FastQC v0.11.2	Open source	http://www.bioinformatics.babraham.ac.uk/projects/fastqc/
Trimmomatic v0.36	Open source	http://www.usadellab.org/cms/?page=trimmomatic
Bowtie2 v2.3.2	Open source	http://bowtie-bio.sourceforge.net/bowtie2/index.shtml
SAMtools v1.5	Open source	http://www.htslib.org/
HISAT2 v2.1.0	Open source	https://daehwankimlab.github.io/hisat2/
StringTie v1.3.3b	Open source	https://ccb.jhu.edu/software/stringtie/
clusterProfiler	R package	https://bioconductor.org/packages/release/bioc/html/clusterProfiler.html
Limma v3.52.4	R package	https://bioconductor.org/packages/release/bioc/html/limma.html
pheatmap	R package	https://cran.r-project.org/web/packages/pheatmap/index.html
GseaVis	R package	https://github.com/junjunlab/GseaVis
ggplot2	R package	https://ggplot2.tidyverse.org/

**Other**		

Powerpac Basic	Bio-rad	164-5050
Agilent 7850 ICP-MS	Agilent Technologies	7850
Inverted microscope	Leica	Leica DMi1
Fluorescence inversion microscope	Nikon	Nikon-Ti2-U
CO2 constant temperature incubator	ESCO	CLM-170B-8-CN
Biosafety cabinet	ESCO	AC2-4S8-CN
Gel Imaging System	Bio-rad	ChemiDoc™ MP Imaging System
Confocal microscope	Zeiss	LSM900
Water purifier	Ro-di	RODI-220B1
Pathological slice scanner	TissueGnostics	TissueFAXS Imaging
Oscillating constant temperature metal bath	Thermo	TUS-200P
Analytical balance	OHAUS	EX623ZH
Refrigerated centrifuge	Eppendorf	5425R
4°C refrigerator	Meiling	YC-525L
-20°C refrigerator	Haier	DW-40L508J
-80°C refrigerator	CryoCube	CryoCube F570
Flow cytometry	BD	FACSMelody
Water bath	BLUEPARD	BWS-27
Ultraviolet spectrophotometer	Tecan	infinite 200pro
Centrifuge	Thermo	Sorvall ST16R

### Resource availability

#### Lead contact

Further information and requests for resources and reagents should be directed to and will be fulfilled by the lead contact, Porf. Ling Qi (qiling1718@gzhmu.edu.cn).

#### Materials availability

This study did not generate new unique reagents.

#### Data and code availability

• RNA-seq data have been deposited to the Gene Expression Omnibus (GEO) database (https://www.ncbi.nlm.nih.gov/geo/) under the accession number GSE256455 and are publicly available as of the date of publication.
• This paper does not report original code.
• Any additional information required to reanalyze the data reported in this paper is available from the lead contact upon request.

### Experimental model and study participant details

#### Cell line

All cell lines used were obtained from the American Type Culture Collection (ATCC). The cells were cultured in Dulbecco's modified Eagle's medium (DMEM) supplemented with 10% fetal bovine serum (FBS) in an incubator at 37°C with 5% CO_2_.

#### Animals

Five-week-old male nude mice were purchased from GemPharmatech Co., Ltd. After a 2-week quarantine, formal experiments were conducted. The mice were divided into three groups, with six mice in each group, for the xenograft tumor experiments. All experiments involving animals were in accordance with institutional animal welfare guidelines and were approved by the Experimental Animal Ethics Committee of Qingyuan People's Hospital (Approval Number: LAEC-2021-028).

### Method details

#### Cell viability assay

Cells in good growth condition were digested with trypsin, centrifuged, resuspended, and counted using a hemocytometer. Cells were seeded uniformly in a 96-well plate at 8,000 cells per well and cultured at 37°C with 5% CO_2_ for 24 hours. After 24 hours, 100 μL of medium containing varying concentrations of EB or ES was added to each well and incubated for another 24 hours. The medium was then discarded, and 100 μL of medium containing 10% CCK-8 solution was added, followed by a 1-hour incubation at 37°C. Absorbance was measured at 450 nm.

#### Colony formation assay

PANC-1 and MiaPaCa-2 human pancreatic cancer cells in good growth condition were digested, centrifuged, resuspended, and counted using a hemocytometer. Cells were then uniformly seeded into 6-well plates at 1,000 cells per well and cultured at 37°C with 5% CO_2_ for 24 hours. The medium was removed, and medium containing different concentrations of EB was added, mixed well, and then placed back in the incubator at 37°C with 5% CO_2_ for another 24 hours. After this period, the medium was replaced with fresh complete medium, which was changed every three days. Culturing was stopped when visible cell colonies were observed. Post-cultivation, the medium was removed, the wells were washed with PBS, fixed with 4% paraformaldehyde for 20 minutes, stained with 1% crystal violet for 5 minutes, and then washed three times with PBS before drying at room temperature. Finally, the colonies were scanned, photographed, and analyzed.

#### Cell proliferation assay

PANC-1 and MiaPaCa-2 human pancreatic cancer cells, in good growth condition, were digested with trypsin, centrifuged, resuspended, and counted. Cells were seeded at 20,000 per well in 48-well plates and cultured at 37°C with 5% CO_2_ for 24 hours. Medium was replaced with 400 μl of medium containing various concentrations of EB and incubated for 24 hours. An EdU medium of 50 μM was prepared, and 200 μl was added to each well for a 2-hour incubation at 37°C. After discarding the medium, cells were washed twice with PBS and fixed with 4% paraformaldehyde for 30 minutes at room temperature. Glycine solution (2 mg/mL) was added at 200 μl per well and incubated on a shaker at room temperature for 5 minutes. Cells were washed twice with PBS on a shaker for 5 minutes each. Cells were then permeabilized with 200 μl of 0.5% Triton X-100 in PBS for 10 minutes on a shaker. After two additional PBS washes, Apollo® staining solution was added at 200 μl per well and incubated in the dark on a shaker at room temperature for 30 minutes. Cells were washed twice with 0.5% Triton X-100 in PBS for 10 minutes each and twice with PBS for 5 minutes each. Hoechst 33342 staining solution was added, and cells were incubated in the dark on a shaker for 30 minutes. After a final wash with PBS, cells were observed under a fluorescence microscope. Five random fields were chosen for image capture, counting, and analysis.

#### Cell scratch assay

PANC-1 and MiaPaCa-2 human pancreatic cancer cells were enzymatically dissociated with trypsin, centrifuged, resuspended, and counted using a hemocytometer before seeding into scratch chambers. Cells were grown to near confluence before chamber removal, followed by two washes with 1x PBS and documentation via photography. Cells were then treated for 24 hours with medium containing varying concentrations of EB, after which the medium was replaced with complete growth medium. Photographs were taken again at 24 hours. Three replicates per concentration were set up. The images were analyzed with ImageJ software to measure the scratch distance, and the results were used to calculate the migration distances of the cancer cells.

#### Transwell invasion assay

Matrigel was thawed overnight at 4°C and then transferred to an ice box before the experiment. The gel was mixed thoroughly using pre-cooled pipette tips. A defined volume of diluted Matrigel was added vertically to the upper chamber of a Transwell and spread evenly across the bottom. This was incubated at 37°C with 5% CO_2_ for 3 hours to allow the Matrigel to polymerize into a film. Excess liquid was removed from the upper chamber after incubation, and each well received 100 μL of serum-free medium to hydrate the basement membrane for another 30 minutes. After removing the liquid from the upper chamber and ensuring no liquid had passed through to the lower chamber, cells were ready to be seeded. Logarithmically growing cells were dissociated with trypsin, resuspended in serum-free medium, counted, and seeded in the Transwell upper chamber at 2×10^4^ cells per 100 μL. The upper chamber contained serum-free medium, while the lower chamber was filled with 600 μL of complete medium. After 6 hours, varying concentrations of EB were added to both chambers—the upper contained serum-free medium with EB, and the lower contained complete medium with the compound. The treatment lasted for 24 hours. Post-treatment, the medium was discarded, wells were washed twice with 1x PBS, and cells were fixed with 4% paraformaldehyde for 30 minutes. Non-invaded cells in the upper chamber were removed with a cotton swab, and the membrane was stained with 1% crystal violet for 5 minutes. After washing off the stain with PBS, the membranes were dried invertedly and the invaded cells were counted for data analysis.

#### Western blot

Tissue/cell protein extracts were prepared with lysis buffer (Beyotime, P0013) containing 1x proteasome inhibitor (KeyGEN, KGP603) and 1 mM PMSF (Beyotime, ST506). After centrifugation at 12,000 rpm for 10 min at 4°C, supernatants of cell lysates were obtained and protein concentration was measured by using the BCA kit (Thermo, #23227). Proteins were separated by SDS-PAGE and transferred to PVDF membranes (Mili, #IPVH00010). The membrane was blocked with 5% nonfat-milk in 1x TBST for 1 h and incubated overnight at 4°C with a 1:1000 dilution of primary antibody. Subsequently, the HRP-conjugated secondary antibody was then used at a 1:5000 dilution for 1 h. Scan, image, and analyze using a chemiluminescence imaging system.

#### RNA-seq

PANC-1 pancreatic cancer cells were treated with DMSO/10 μM EB for 24 hours (four biological replicates per group, n=4), then washed twice with PBS. The supernatant was discarded, and cells were lysed with 1 mL of Trizol, with repeated pipetting for complete dissolution. The clear lysate was then transferred into RNase-free cryovials, flash-frozen in liquid nitrogen, and stored at -80°C. The samples were immediately sent to Sangon Biotech (Shanghai) Co., Ltd. for transcriptomic RNA sequencing, with triplicates for each group. RNA was harvested using the Rneasy mini plus kit (Qiagen). 1.4 μg of total RNA was used for the construction of sequencing libraries. RNA libraries for RNA-seq were prepared using the Hieff NGS™ MaxUp Dual-mode mRNA Library Prep Kit for Illumina following the manufacturer’s protocols. The library strategy was RNA-Seq, the library source was transcriptomic, and the library selection was cDNA. The sequencing was performed using the Illumina NovaSeq 6000 instrument.

Data processing was conducted using CLC Genomics Workbench v 11.0.1. Sequence reads were trimmed for adaptor sequence and low-quality sequence using CLC Genomics Workbench (parameter - Quality limit: 0.05). Trimmed sequence reads were mapped to GrCM38 using CLC Genomics Workbench (parameters - mismatch cost: 2, insertion cost: 3, deletion cost: 3, length fraction: 0.8, similarity fraction: 0.8). Read count extraction and normalization were performed using CLC Genomics Workbench. The assembly used was hg38. Additional tools used in the data processing included Trimmomatic v0.36, FastQC v0.11.2, Bowtie2 v2.3.2, SAMtools v1.5, HISAT2 v2.1.0 and StringTie v1.3.3b. Comparative analysis was conducted on the transcriptomic alterations in the pancreatic cancer cells before and after EB treatment to identify differentially expressed genes and investigate the underlying mechanisms of EB action on pancreatic cancer. Sequencing data was processed using R version 4.2.1, with the KEGG analysis conducted utilizing the clusterProfiler^31^ package.

#### Nude mouse xenograft tumor model

Balb/c nude mice, SPF grade, male, were housed under conditions of 22±1°C temperature, 40-70% humidity, and a 12-hour light-dark cycle. PANC-1 pancreatic cancer cells were cultured and, upon reaching 80-90% confluence, were harvested, centrifuged, and resuspended in PBS to a concentration of 5×10^7^ cells/mL. Each mouse was injected subcutaneously in the right flank with 200 μL of cell suspension (1×10^7^ cells per mouse) to induce tumor formation. Once tumors were established, mice were randomized into control and treatment groups to begin administration of drugs. EB was dissolved in saline and injected intraperitoneally daily at 20mg/kg or 40mg/kg concentrations. Tumor growth and mouse conditions were monitored throughout the experiment. After 4 weeks, mice were euthanized, tumor volumes measured, and tumors excised and weighed to evaluate the therapeutic effect of EB on pancreatic cancer.

#### Immunohistochemical and H&E staining

Tissue sections of 2-4 microns were baked at 70°C for one hour or overnight. After removing from the oven, sections were dewaxed to water through a series of xylene I, II, III, IV, V each for 5 minutes, anhydrous ethanol I, II each for 3 minutes, 95% ethanol for 3 minutes, and washed thrice with distilled water. EDTA antigen retrieval was then conducted by placing sections in EDTA solution and microwaving on high for 11 minutes and medium for 4 minutes, followed by cooling to room temperature. Sections were washed three times on a shaker with PBST buffer, each for 5 minutes. A 3% hydrogen peroxide solution was applied for 10 minutes. Sections were again washed three times on a shaker with PBST buffer, each for 5 minutes. After wiping the liquid around the tissue, primary antibody was added and sections were incubated overnight at 4°C in a humidified chamber. The primary antibody was removed, sections were briefly washed with distilled water, followed by three washes on a shaker with PBST buffer, each for 5 minutes. Secondary antibody was added (following the same requirements as the primary) and incubated for 30 minutes at a constant temperature. Sections were washed briefly with distilled water and then washed three times on a shaker with PBST buffer, each for 5 minutes. DAB chromogen was applied for about 5 minutes followed by washing with water, counterstained with hematoxylin for 1 minute, and blued. Dehydration through graded alcohols, cleared in xylene, and mounted with neutral balsam.

#### ROS/apotosis assay

Well-growing PANC-1 and MiaPaCa-2 human pancreatic cancer cells were centrifuged, resuspended, and counted using a hemocytometer. Cells were seeded in 6-well plates at 3.5×10^5^ cells per well. The next day, cells were treated for 24 hours with medium containing varying concentrations of EB, followed by probe loading. A 10 mM stock solution of H2DCFDA was diluted with PBS to prepare a working solution with a final concentration of 10 μM. The culture medium was removed, cells were washed twice with PBS, and then the diluted H2DCFDA working solution was added, with 1 mL per well in a 6-well plate. Cells were incubated for 30 minutes at 37°C, the H2DCFDA solution was removed, and cells were washed twice with PBS. Cells were then digested with trypsin, observed under a microscope, digestion was terminated with serum-free medium, and cells were centrifuged at 1000 rpm for 3 minutes. Cells were washed twice with PBS to fully remove any H2DCFDA that did not enter the cells. Finally, cells were resuspended in 500 μL PBS and analyzed using flow cytometry. For apoptosis detection, reagents from the Annexin V-FITC/PI double staining kit were used.

#### SiRNA transfection

SiRNA transfection was performed on well-growing PANC-1 and MiaPaCa-2 human pancreatic cancer cells. Cells were digested with trypsin, centrifuged, resuspended, and counted using a hemocytometer. Cells were seeded at 200,000 per well in 6-well plates and cultured at 37°C with 5% CO_2_ for 24 hours. After 24 hours, transfection complexes were prepared by adding 250 μl of OPTI-MEM and 5 μl of Lipofectamine 3000 reagent to a sterile 1.5 ml centrifuge tube, gently mixed, and incubated at room temperature for 5 min. Simultaneously, in another sterile 1.5 ml centrifuge tube, 250 μl of OPTI-MEM was mixed with 5 μl of siRNA, which was dissolved according to the manufacturer's instructions. After another 5 min, the transfection reagent-medium mixture was added dropwise to the siRNA-medium mixture, gently mixed, and incubated at room temperature for 20 min. During this incubation, the medium in the 6-well plates was replaced with 1.5 ml of serum-free medium per well. After 20 min, 500 μl of the transfection mixture was added to each well for a final volume of 2 ml. The plates were gently rocked to distribute the complexes evenly, followed by incubation in a 37°C, 5% CO_2_ incubator, and the medium was replaced with complete medium after 6h. Protein expression at 48-96 hours post-transfection.

#### Cell copper colorimetric assay (complexing method)

PANC-1 and MiaPaCa-2 human pancreatic cancer cells were seeded appropriately into 10 cm dishes and treated the next day with culture medium containing different concentrations of EB for 24 h, after which cells were collected. A Cell Copper Colorimetric Assay Kit (Cat#E-BC-K775-M-96T, Elabscience) was used according to the manufacturer's instructions. The optical density (OD) at 580 nm was measured using a microplate reader. A standard curve was plotted using the OD values and copper content of the standards, and the copper ion concentration in the samples was calculated based on the standard curve.

#### Measurement of intracellular copper ion content by ICP-MS

Well-growing PANC-1 and MiaPaCa-2 human pancreatic cancer cells were centrifuged, resuspended, and counted using a hemocytometer. Cells were seeded in 6-well plates at 3.5×10^5^ cells per well. The next day, cells were treated for 24 hours with medium containing varying concentrations of EB. After EB and control treatments, cells were washed with PBS, digested, centrifuged, counted, and resuspended in 10 ml of deionized water for analysis. The Agilent 7850 ICP-MS was used to measure intracellular copper ion content in pancreatic cancer cells. The instrument parameters included 1550 W power, 1.08 L/min nebulizer gas, and 15 L/min plasma gas. The copper ion content was normalized to cell counts to determine the intracellular concentration for each sample.

#### Measurement of intracellular copper ion content by fluorescent imaging

Well-growing PANC-1 and MiaPaCa-2 human pancreatic cancer cells were centrifuged, resuspended, and counted using a hemocytometer. Cells were seeded in 6-well plates at 3.5×10^5^ cells per well. The next day, cells were treated for 24 hours with medium containing varying concentrations of EB. After EB and control treatments 24 hours, cells were washed with PBS and stained with CS1(Coppersensor-1, Cat#HY-141511, MCE), a membrane-permeable fluorescent dye, at a final concentration of 5 μM. Cells were incubated in the dark for 20 minutes and then imaged using a fluorescence microscope with 543 nm excitation. This method provided visualization and quantification of intracellular copper ion levels.

### Quantification and statistical analysis

All results are reported as mean ± SD for triplicate experiments unless otherwise noted. Data were analyzed using GraphPad Prism V8.0.2 statistical software. Two groups were compared with Prism software using a two-tailed unpaired Student’s t-test. A p-value of less than 0.05 was considered to indicate statistical significance (∗: *p* < 0.05, ∗∗: *p* < 0.01, ∗∗∗: *p* < 0.001).

## References

[1] Siegel R.L., Miller K.D., Wagle N.S., Jemal A. (2023). Cancer statistics, 2023. CA. Cancer J. Clin..

[2] Park W., Chawla A., O'Reilly E.M. (2021). Pancreatic Cancer: A Review. JAMA.

[3] Springfeld C., Ferrone C.R., Katz M.H.G., Philip P.A., Hong T.S., Hackert T., Büchler M.W., Neoptolemos J. (2023). Neoadjuvant therapy for pancreatic cancer. Nat. Rev. Clin. Oncol..

[4] Cech N.B., Oberlies N.H. (2023). From plant to cancer drug: lessons learned from the discovery of taxol. Nat. Prod. Rep..

[5] Atanasov A.G., Zotchev S.B., Dirsch V.M., Supuran C.T., Supuran C.T. (2021). Natural products in drug discovery: advances and opportunities. Nat. Rev. Drug Discov..

[6] Yang B., Zhao Y., Lou C., Zhao H. (2016). Eupalinolide O, a novel sesquiterpene lactone from Eupatorium lindleyanum DC., induces cell cycle arrest and apoptosis in human MDA-MB-468 breast cancer cells. Oncol. Rep..

[7] Hu H., Bai H., Huang L., Yang B., Zhao H. (2023). Eupalinolide J Inhibits Cancer Metastasis by Promoting STAT3 Ubiquitin-Dependent Degradation. Molecules.

[8] Zhang Y., Dong F., Cao Z., Wang T., Pan L., Luo W., Ding W., Li J., Jin L., Liu H. (2022). Eupalinolide A induces autophagy via the ROS/ERK signaling pathway in hepatocellular carcinoma cells *in vitro* and *in vivo*. Int. J. Oncol..

[9] Tian S., Chen Y., Yang B., Lou C., Zhu R., Zhao Y., Zhao H. (2018). F1012-2 inhibits the growth of triple negative breast cancer through induction of cell cycle arrest, apoptosis, and autophagy. Phytother Res..

[10] Yang B., Shen J.W., Zhou D.H., Zhao Y.P., Wang W.Q., Zhu Y., Zhao H.J. (2019). Precise discovery of a STAT3 inhibitor from Eupatorium lindleyanum and evaluation of its activity of anti-triple-negative breast cancer. Nat. Prod. Res..

[11] Yang L., Chen H., Hu Q., Liu L., Yuan Y., Zhang C., Tang J., Shen X. (2022). Eupalinolide B attenuates lipopolysaccharide-induced acute lung injury through inhibition of NF-κB and MAPKs signaling by targeting TAK1 protein. Int. Immunopharmacol..

[12] Yan G., Ji L., Luo Y., Hu Y. (2011). Antioxidant activities of extracts and fractions from Eupatorium lindleyanum DC. Molecules.

[13] Chu C., Yao S., Chen J., Wei X., Xia L., Chen D., Zhang J. (2016). Eupatorium lindleyanum DC. flavonoids fraction attenuates lipopolysaccharide-induced acute lung injury in mice. Int. Immunopharmacol..

[14] Chu C., Ren H., Xu N., Xia L., Chen D., Zhang J. (2016). Eupatorium lindleyanum DC. sesquiterpenes fraction attenuates lipopolysaccharide-induced acute lung injury in mice. J. Ethnopharmacol..

[15] Tsvetkov P., Coy S., Petrova B., Dreishpoon M., Verma A., Abdusamad M., Rossen J., Joesch-Cohen L., Humeidi R., Spangler R.D. (2022). Copper induces cell death by targeting lipoylated TCA cycle proteins. Science.

[16] Yang Y., Li M., Chen G., Liu S., Guo H., Dong X., Wang K., Geng H., Jiang J., Li X. (2023). Dissecting copper biology and cancer treatment: ‘Activating Cuproptosis or suppressing Cuproplasia’. Coord. Chem. Rev..

[17] Yang Y., Liang S., Geng H., Xiong M., Li M., Su Q., Jia F., Zhao Y., Wang K., Jiang J. (2022). Proteomics revealed the crosstalk between copper stress and cuproptosis, and explored the feasibility of curcumin as anticancer copper ionophore. Free Radic. Biol. Med..

[18] Zulkifli M., Spelbring A.N., Zhang Y., Soma S., Chen S., Li L., Le T., Shanbhag V., Petris M.J., Chen T.-Y. (2023). FDX1-dependent and independent mechanisms of elesclomol-mediated intracellular copper delivery. Proc. Natl. Acad. Sci. USA.

[19] Matsushita M., Nakamura T., Moriizumi H., Miki H., Takekawa M. (2020). Stress-responsive MTK1 SAPKKK serves as a redox sensor that mediates delayed and sustained activation of SAPKs by oxidative stress. Sci. Adv..

[20] Wada T., Stepniak E., Hui L., Leibbrandt A., Katada T., Nishina H., Wagner E.F., Penninger J.M. (2008). Antagonistic control of cell fates by JNK and p38-MAPK signaling. Cell Death Differ..

[21] Gao J., Wu X., Huang S., Zhao Z., He W., Song M. (2023). Novel insights into anticancer mechanisms of elesclomol: More than a prooxidant drug. Redox Biol..

[22] Xue Q., Kang R., Klionsky D.J., Tang D., Liu J., Chen X. (2023). Copper metabolism in cell death and autophagy. Autophagy.

[23] Pan Q., Kleer C.G., van Golen K.L., Irani J., Bottema K.M., Bias C., De Carvalho M., Mesri E.A., Robins D.M., Dick R.D. (2002). Copper deficiency induced by tetrathiomolybdate suppresses tumor growth and angiogenesis. Cancer Res..

[24] Mandinov L., Mandinova A., Kyurkchiev S., Kyurkchiev D., Kehayov I., Kolev V., Soldi R., Bagala C., de Muinck E.D., Lindner V. (2003). Copper chelation represses the vascular response to injury. Proc. Natl. Acad. Sci. USA.

[25] Prohaska J.R. (2008). Role of copper transporters in copper homeostasis. Am. J. Clin. Nutr..

[26] Yamashita Y., Ikeda T., Matsuda M., Maji D., Hoshino T., Mizushima T. (2012). Purification and characterization of HSP-inducers from Eupatorium lindleyanum. Biochem. Pharmacol..

[27] Zhang Y., Zhou Q., Lu L., Su Y., Shi W., Zhang H., Liu R., Pu Y., Yin L. (2023). Copper Induces Cognitive Impairment in Mice via Modulation of Cuproptosis and CREB Signaling. Nutrients.

[28] Li Y., Du Y., Zhou Y., Chen Q., Luo Z., Ren Y., Chen X., Chen G. (2023). Iron and copper: critical executioners of ferroptosis, cuproptosis and other forms of cell death. Cell Commun. Signal..

[29] Kirshner J.R., He S., Balasubramanyam V., Kepros J., Yang C.-Y., Zhang M., Du Z., Barsoum J., Bertin J. (2008). Elesclomol induces cancer cell apoptosis through oxidative stress. Mol. Cancer Ther..

[30] Zheng P., Zhou C., Lu L., Liu B., Ding Y. (2022). Elesclomol: a copper ionophore targeting mitochondrial metabolism for cancer therapy. J. Exp. Clin. Cancer Res..

[31] Yu G., Wang L.-G., Han Y., He Q.-Y. (2012). clusterProfiler: an R package for comparing biological themes among gene clusters. OMICS.

