# Supplementary material for: Eupalinolide B suppresses pancreatic cancer by ROS generation and potential cuproptosis
Source: iScience. 2024 Jul 14;27(8):110496. doi: 10.1016/j.isci.2024.110496 (PMC11295471; doi:10.1016/j.isci.2024.110496)

iScience, Volume *27*

Supplemental information

Eupalinolide B suppresses pancreatic cancer by ROS generation and potential cuproptosis

Qingtian Huang, Jie Yang, Jiaxing Zhang, Leyi Yao, Baoyi Jiang, Siyuan Du, Fengjin Li, Qian Peng, Lingsha Qin, Yanfen Wang, and Ling Qi

Figure S1. Molecular and Pathway Analyses of EB-Treated Pancreatic Cancer Cells, Related to Figure 3. (A) Fluorescent imaging of ROS in MiaPaCa-2 and PANC-1 cells induced by EB. (B) KEGG pathway enrichment of up-regulated genes between EB-treated and control groups. (C) KEGG pathway enrichment of down-regulated genes between EB-treated and control groups. (D) Changes in MAPK pathway-related proteins following administration of various doses of EB. (E) Protein alterations post-inhibition of JNK phosphorylation by SP600125. (F) Changes in cell viability following JNK phosphorylation inhibition by SP600125. (G) Ferroptosis-associated proteins following 24 hours of treatment with varying concentrations of EB. "ns" indicates no statistical significance in p-value.

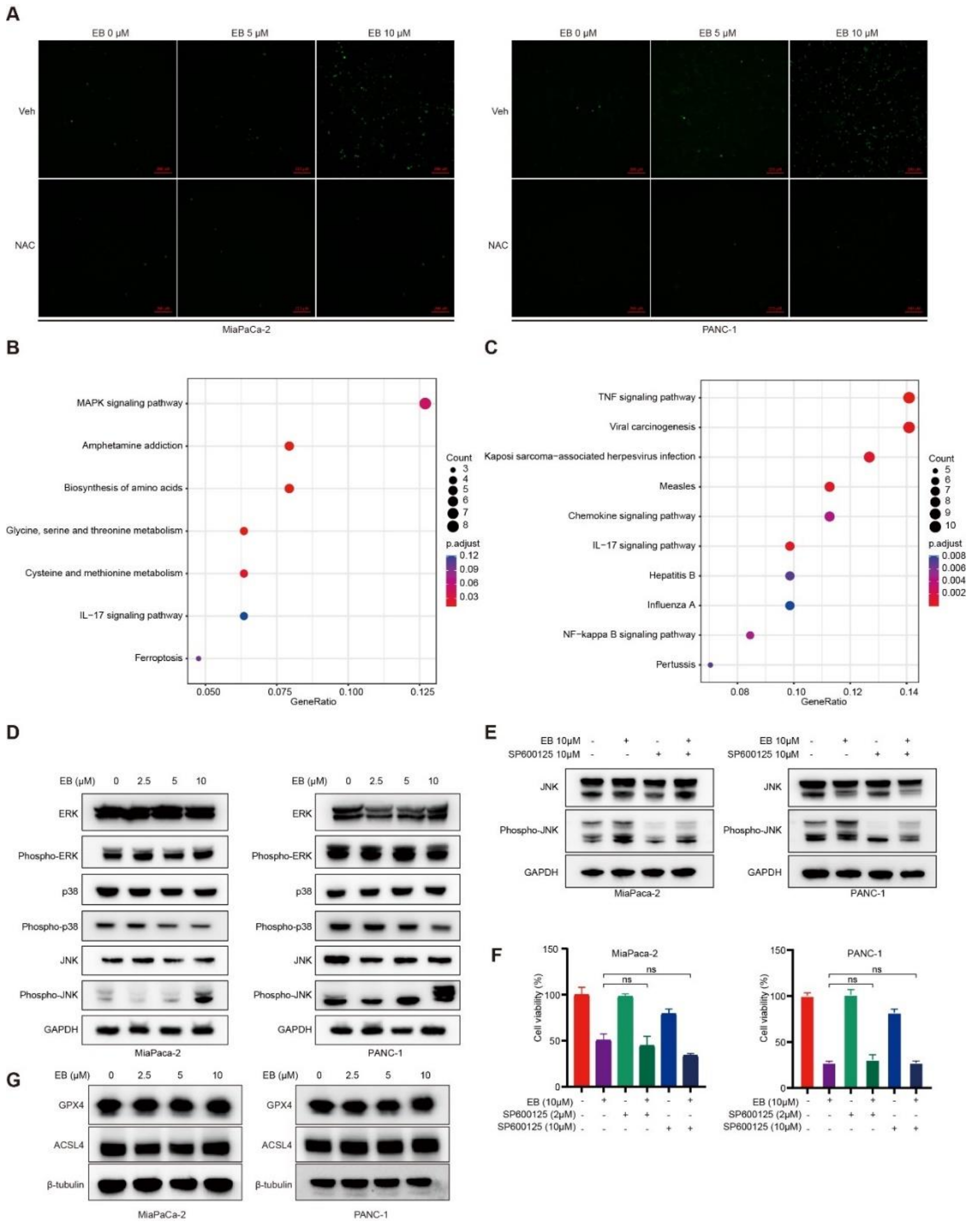

Figure S2. Analysis of Apoptosis in EB-Treated Pancreatic Cancer Cells, Related to Figure 3. (A) Apoptotic proteins following 24 hours of treatment with varying concentrations of EB. (B) Flow cytometry detection of reactive oxygen species (ROS) effects in MiaPaCa-2 and PANC-1 cells treated with EB. (C) Fluorescent imaging of apoptosis in MiaPaCa-2 and PANC-1 cells induced by EB.

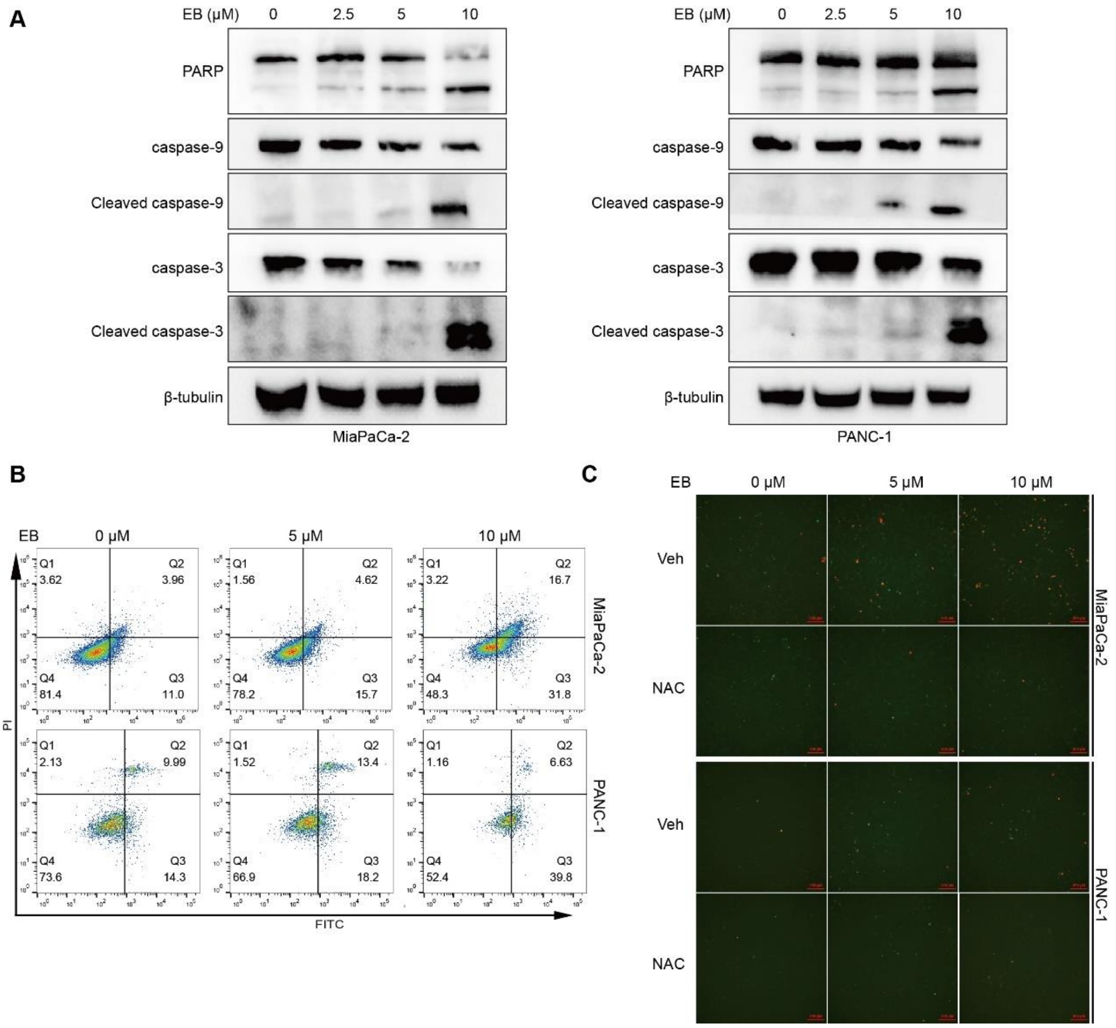

Figure S3. Detection of Intracellular Copper Ion Content Using Coppersensor-1, Related to Figure 4. (A) Fluorescent imaging of Copper in MiaPaCa-2 cells treated with different concentrations of EB. (B) Fluorescent imaging of Copper in PANC-1 cells treated with different concentrations of EB.

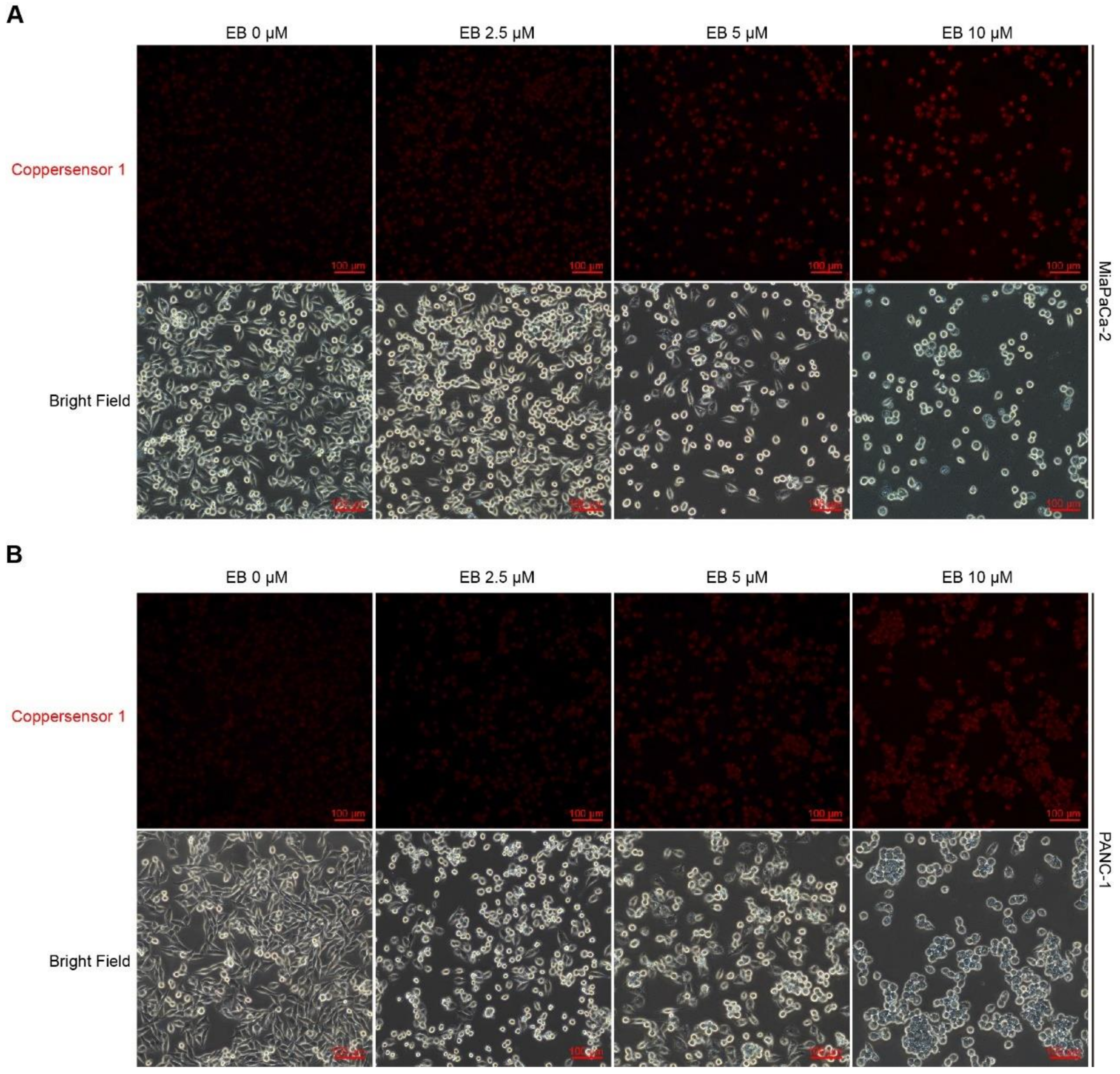

Supplement: Document S1. Figures S1–S3 [file mmc1.pdf]
